# A nucleotide binding–independent role for γ-tubulin in microtubule capping and cell division

**DOI:** 10.1083/jcb.202204102

**Published:** 2023-01-25

**Authors:** Adi Y. Berman, Michal Wieczorek, Amol Aher, Paul Dominic B. Olinares, Brian T. Chait, Tarun M. Kapoor

**Affiliations:** 1 Laboratory of Chemistry and Cell Biology, The Rockefeller University, New York, NY, USA; 2 Tri-Institutional PhD Program in Chemical Biology, The Rockefeller University, New York, NY, USA; 3 Laboratory of Mass Spectrometry and Gaseous Ion Chemistry, The Rockefeller University, New York, NY, USA

## Abstract

The γ-tubulin ring complex (γ-TuRC) has essential roles in centrosomal and non-centrosomal microtubule organization during vertebrate mitosis. While there have been important advances in understanding γ-TuRC-dependent microtubule nucleation, γ-TuRC capping of microtubule minus-ends remains poorly characterized. Here, we utilized biochemical reconstitutions and cellular assays to characterize the human γ-TuRC’s capping activity. Single filament assays showed that the γ-TuRC remained associated with a nucleated microtubule for tens of minutes. In contrast, caps at dynamic microtubule minus-ends displayed lifetimes of ∼1 min. Reconstituted γ-TuRCs with nucleotide-binding deficient γ-tubulin (γ-tubulin^ΔGTP^) formed ring-shaped complexes that did not nucleate microtubules but capped microtubule minus-ends with lifetimes similar to those measured for wild-type complexes. In dividing cells, microtubule regrowth assays revealed that while knockdown of γ-tubulin suppressed non-centrosomal microtubule formation, add-back of γ-tubulin^ΔGTP^ could substantially restore this process. Our results suggest that γ-TuRC capping is a nucleotide-binding-independent activity that plays a role in non-centrosomal microtubule organization during cell division.

## Teaser

Berman et al. examine the capping activity of the γ-TuRC. Using in vitro and cellular assays, they characterize how the γ-TuRC's nucleotide-independent capping activity regulates microtubule dynamics and how it contributes to microtubule formation during cell division.

## Introduction

During mitosis, the formation and organization of non-centrosomal microtubules, such as those generated by chromosome-dependent microtubule nucleation, contribute to the assembly of the bipolar metaphase spindle (Heald and Khodjakov, 2015; Meunier and Vernos, 2016; Kapoor, 2017). Cell biology studies have demonstrated that the γ-tubulin ring complex (γ-TuRC) plays a critical role in these pathways (Akhmanova and Kapitein, 2022). Depletion of γ-TuRC components in various model systems, including *Caenorhabditis elegans*, *Saccharomyces cerevisiae*, *Drosophila melanogaster*, and cultured human cells, results in the loss of spindle bipolarity (Strome et al., 2001; Hannak et al., 2002; Mahoney et al., 2006; McKinley and Cheeseman, 2017; Cota et al., 2017). Moreover, microtubule regrowth assays in cells depleted of γ-TuRC proteins show either complete or significant loss of non-centrosomal microtubule formation, while centrosomal microtubules form, albeit more slowly (Hannak et al., 2002; Cota et al., 2017; Tsuchiya and Goshima, 2021). While the loss of γ-TuRC-dependent microtubule nucleation could be responsible for these phenotypes, it is possible that other activities of this complex may also be important. Specifically, biochemical assays have suggested that the γ-TuRC acts as a cap to suppress the addition or loss of tubulin subunits at the microtubule minus-end (Wiese and Zheng, 2000). Capping by the γ-TuRC can occur following a nucleation event where the γ-TuRC remains associated with the newly nucleated microtubule or when the γ-TuRC binds to the free minus-end of an existing microtubule (Wiese and Zheng, 2000). However, we do not understand the role of γ-TuRC’s capping activity in microtubule formation and organization in cells.

Cryo-EM structures of the *Xenopus* and human γ-TuRC have revealed that the complex is an asymmetric, cone-shaped assembly (Liu et al., 2020; Consolati et al., 2020; Wieczorek et al., 2020a). The most abundant γ-TuRC component, γ-tubulin, is positioned at the top of the cone, where it can mediate interactions with α,β-tubulin dimers. The asymmetric cone is composed of seven Y-shaped subunits, four of which are comprised of the γ-tubulin small complex (γ-TuSC) proteins γ-tubulin, GCP2 and 3, and three γ-TuSC-like Y-shaped subunits, consisting of γ-tubulin bound to GCP4, 5, or 6 (Oegema et al., 1999; Murphy et al., 2001; Kollman et al., 2010). Across the interior of the cone resides a luminal bridge, which is composed of the N-terminal domains of GCP6 and GCP3 associated with actin and MZT1 proteins, while MZT2 interacts with the outer face of the cone (Wieczorek et al., 2020b; Würtz et al., 2022). These findings from structural studies, along with additional biochemical data, have facilitated analyses of recombinant γ-TuRC and the basis of the γ-TuRC’s asymmetric organization (Zimmermann et al., 2020; Wieczorek et al., 2021; Würtz et al., 2021). Reconstitution studies thus far have predominantly focused on the γ-TuRC’s nucleation activity (Zimmermann et al., 2020; Wieczorek et al., 2021; Würtz et al., 2021), which is retained in a partial γ-TuRC complex lacking the luminal bridge (γ-TuRC^ΔLB^; Wieczorek et al., 2021). Additionally, GTP binding by γ-tubulin has been found to be important for γ-TuRC-mediated microtubule nucleation (Gombos et al., 2013; Wieczorek et al., 2021). A point mutation in γ-tubulin’s nucleotide-binding pocket (N229A), which reduces the yeast γ-tubulin’s affinity for nucleotide by approximately three orders of magnitude, compromised microtubule nucleation activity in yeast γ-TuSCs and the partial human complex γ-TuRC^ΔLB^ (Gombos et al., 2013; Wieczorek et al., 2021). However, the dependence of the γ-TuRC’s microtubule capping activity on nucleotide binding by γ-tubulin is not known.

Here, we examine the microtubule minus-end capping activity of the γ-TuRC. We find that the association of γ-TuRCs with microtubule minus-ends following a nucleation event persists over tens of minutes, while caps at dynamic minus-ends have lifetimes of ∼1 min. Nucleotide-binding-deficient γ-TuRC capped dynamic microtubules with similar lifetimes, despite its compromised nucleation activity. Microtubule regrowth assays in mitotic cells revealed that non-centrosomal microtubule formation, which was suppressed in γ-tubulin knockdown cells, is observed in cells expressing nucleotide-binding deficient γ-tubulin. Together, our results suggest that γ-TuRC capping is nucleotide-binding-independent and contributes to non-centrosomal microtubule formation and organization during cell division.

## Results

### γ-TuRC^γ-Tub-WT^ caps nucleated and preformed microtubules

**Figure 1.**
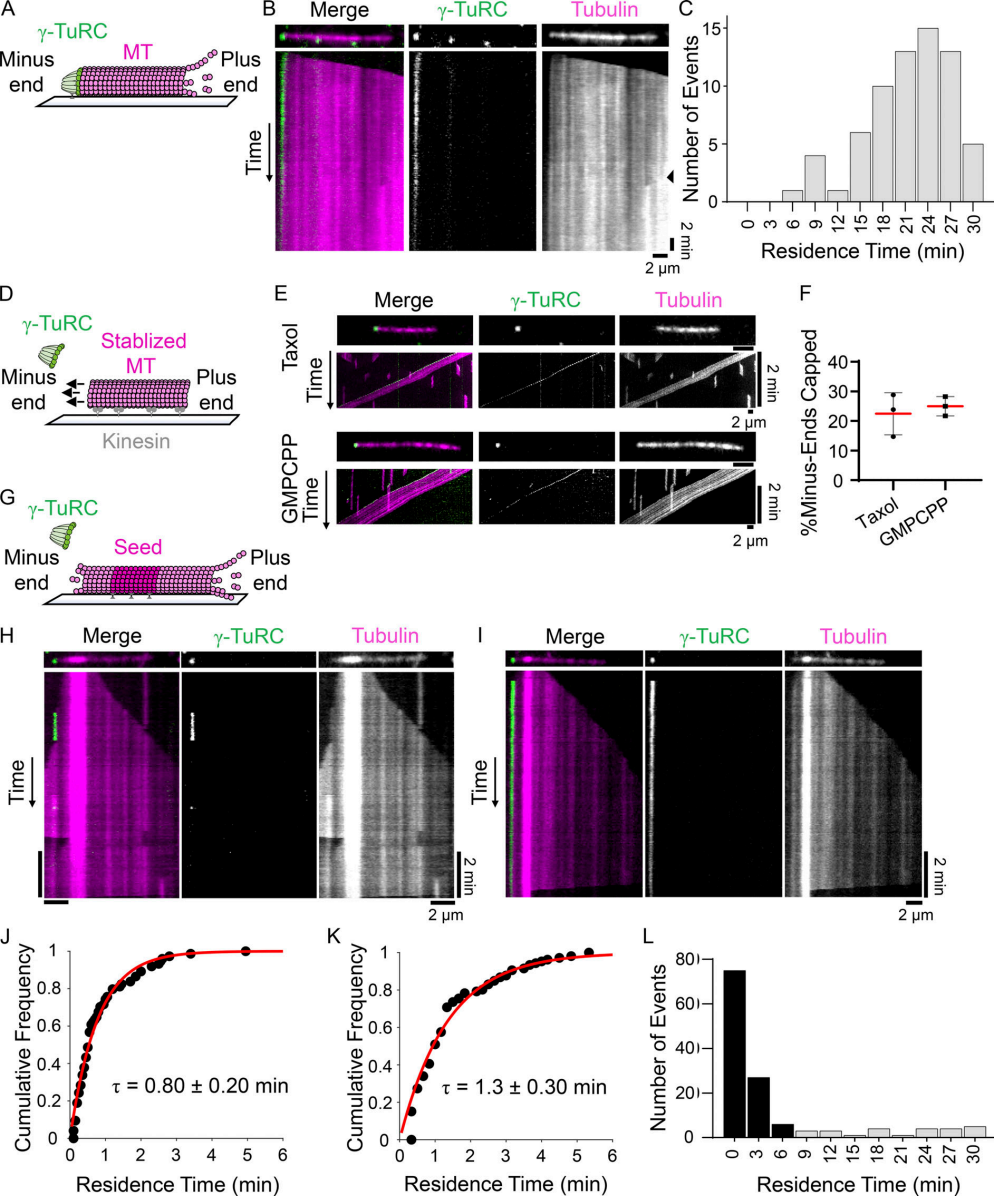
**Recombinant γ-TuRC**^**γ-Tub-WT**^ **caps nucleated microtubules and pre-formed minus-ends. (A)** Schematic of the TIRF-based assay to analyze microtubules nucleated by recombinant γ-TuRC. Surface immobilized GFP-tagged γ-TuRC (green) and polymerized tubulin (pink) are shown. **(B)** Image and kymograph of a microtubule nucleation event from γ-TuRC^γ-Tub-WT^. Two-color overlay of tubulin (magenta) and γ-TuRC^γ-Tub–WT^ (green), and single-channel images are shown. Black triangle (right kymograph) marks signal from the appearance of another polymerizing microtubule nucleated nearby. **(C)** Frequency distribution of the residence times of γ-TuRC^γ-Tub-WT^ at microtubule minus-ends after a nucleation event. Bin size = 3 min, *n* = 67 events, *N* = 3 independent experiments. **(D)** Schematic of the assay to analyze GFP-tagged γ-TuRC (green) capping of stabilized microtubules (pink) bound to surface-immobilized kinesin motor domains (non-fluorescent). Arrows indicate the directional movement of microtubules in the presence of MgATP (100 µM). **(E)** Images and kymographs of γ-TuRC^γ-Tub–WT^ capping taxol- or GMPCPP-stabilized microtubules. Two-color overlay of tubulin (magenta) and γ-TuRC^γ-Tub–WT^ (green), and single channel images are shown. The images and kymographs are shown at different scales. **(F)** Percentage of taxol- or GMPCPP-stabilized microtubule minus-ends capped by γ-TuRC^γ-Tub-WT^ at 3 min from the start of imaging. Mean (red line) and error (SD) are shown. Taxol: *n* = 1,770 total microtubules from *N* = 3 independent experiments. GMPCPP: *n* = 2,326 total microtubules from *N* = 3 independent experiments. **(G)** Schematic of the assay to analyze recombinant γ-TuRC capping dynamic microtubule minus-ends. Biotinylated “bright” GMPCPP seed (magenta, 12.5% X-rhodamine-tubulin), polymerizing “dim” (pink, 2.5% X-rhodamine-tubulin) minus- and plus-end extensions, and GFP-tagged γ-TuRCs (green) are shown. **(H and I)** Images and kymographs of γ-TuRC^γ-Tub–WT^ capping events on dynamic microtubules. Two-color overlay of tubulin (magenta) and γ-TuRC^γ-Tub–WT^ (green), and single-channel images are shown. **(J and K)** Cumulative frequency of the residence times of γ-TuRC^γ-Tub-WT^ capping events where association and dissociation of the cap were observed from short (10 min; J) or long (30 min; K) duration experiments, fitted to a single exponential (red line) with indicated mean residence time, τ. Error = 95% C.I. J: *n* = 74 events (83% of total events), *N* = 3 independent experiments. K: *n* = 107 events (80% of total events) from *N* = 3 independent experiments. **(L)** Frequency distribution of γ-TuRC^γ-Tub-WT^ residence times from longer duration experiments (30 min). Events where γ-TuRC^γ-Tub-WT^ dissociation from minus-ends is observed (black bars) and where γ-TuRC^γ-Tub-WT^ remained associated with minus-ends throughout the course of imaging (gray bars) are plotted. Bin size = 3 min. *n* = 134 total events from *N* = 2 independent experiments. Scale bars: distance (horizontal) = 2 μm, time (vertical) = 2 min.

To examine the association of the γ-TuRC with microtubule minus-ends, recombinant γ-TuRC^γ-Tub-WT^ containing GFP-tagged MZT2 was purified, as described previously (Wieczorek et al., 2021). We first used this complex to perform nucleation assays and characterized the association of the γ-TuRC at the minus-end of newly formed microtubules (Fig. 1 A). Nucleation of microtubules from a single surface-bound γ-TuRC^γ-Tub-WT^ was monitored for up to 30 min, after which photobleaching of the γ-TuRC^γ-Tub-WT^ or overcrowding of the microtubules that formed limited our analyses. For the majority of nucleation events (∼88%; *n* = 76 total events from *N* = 3 independent experiments), the γ-TuRC remained associated with the microtubule for several minutes (range: 7.0–29.8 min; Fig. 1, B and C) and remained associated over the course of the experiment, consistent with previous qualitative analyses (Consolati et al., 2020).

Next, we established a TIRF-based assay to examine the capping of taxol-stabilized microtubules by γ-TuRC^γ-Tub-WT^. Taxol-stabilized microtubules were attached to passivated coverslips to which the plus-end directed kinesin-1 fragment (residues 1-560, K560) was adhered, and their motility in the presence of MgATP (100 µM) was used to determine microtubule polarity and to exclude any non-specifically coverslip-attached γ-TuRC (Fig. 1 D). Following incubation with γ-TuRC^γ-Tub-WT^ (50 pM), we observed 23 ± 7% (*n* = 1,770 total microtubules from *N* = 3 independent experiments) of the taxol-stabilized minus-ends to be capped (Fig. 1, E and F), consistent with previous work (Zheng et al., 1995). We next performed this assay using guanylyl-(α,β)-methylene-diphosphonate (GMPCPP)-stabilized microtubules and found that 25 ± 3% (*n* = 2,326 total microtubules from *N* = 3 independent experiments) of the minus-ends were capped (Fig. 1, E and F). Interestingly, taxol- and GMPCPP-stabilized microtubules have been shown to predominantly have 13 or 14 protofilaments, respectively (Ginsburg et al., 2017; Rai et al., 2021). As the γ-TuRC similarly capped taxol- and GMPCPP-stabilized microtubules, our data suggest that changes in protofilament number (13 vs. 14) do not substantially affect the capping activity of the γ-TuRC under our experimental conditions. Further, the γ-TuRC cap often persisted at stable microtubule minus-ends for >2 min, although this could not be measured quantitatively due to the motility of the microtubules in this assay.

We next examined the interactions of GFP-tagged γ-TuRCs with dynamic microtubules (Fig. 1 G). Surface-bound, GMPCPP-stabilized microtubule “seeds” were incubated with γ-TuRC (10–30 pM) and soluble tubulin (15 µM). Both microtubule ends were observed to grow as “dim” extensions from a “bright” seed, with the minus-ends being identified as the slower polymerizing extensions (Walker et al., 1988). Puncta of γ-TuRC^γ-Tub-WT^ were found to bind dynamic microtubule minus-ends over the course of the experiment (total time: 10 min, imaging interval: 3 s; Fig. 1, H and I). Kymographs of single filaments showed dynamic minus-ends, and binding of the γ-TuRC^γ-Tub-WT^ suppressed growth and shrinkage (Fig. 1, H and I). For the majority (∼83%, *n* = 89 total events from *N* = 3 independent experiments) of these events, the γ-TuRC^γ-Tub-WT^ was found to associate and then dissociate from the minus-end (Fig. 1 H). Dissociation of the γ-TuRC^γ-Tub-WT^ was followed by the resumption of minus-end dynamics (Fig. 1 H). An exponential fit of the cumulative frequency plot for these events provided a mean residence time (τ) of 0.80 ± 0.20 min (95% confidence interval [C.I.], Fig. 1 J). In the remaining capping events (∼17%), the γ-TuRC bound to a minus-end but did not dissociate during the course of the experiment (10 min; Fig. 1 I). We repeated our experiments for longer periods of time (total time: 30 min, interval: 10 s) and found that, again, the majority of events (∼80%) showed both binding and dissociation of the γ-TuRC and an exponential fit of the cumulative frequency of the residence times for these events provided a mean residence time (τ) of 1.30 ± 0.30 min (95% C.I., Fig. 1 K). Furthermore, γ-TuRC dissociation from the minus-end was not observed during the course of this experiment (30 min) for a fraction of the events (∼20%, *n* = 134 total events from *N* = 2 independent experiments). As the full binding and unbinding cycle was not observed, these events were not included in the mean residence time calculation (see Materials and methods). The apparent residence time for all events (black bars: both association and dissociation observed; gray bars: association, but no dissociation observed) from the longer duration experiments are shown using a frequency distribution plot (Fig. 1 L).

Taken together, our findings indicate that the γ-TuRC binds a nucleated microtubule for tens of minutes or a stabilized preformed microtubule for 2 or more minutes, and caps dynamic microtubule minus-ends with lifetimes of ∼1 min.

### Nucleotide-binding deficient γ-tubulin incorporates into native-like γ-TuRCs that cannot nucleate microtubules

**Figure S1.**
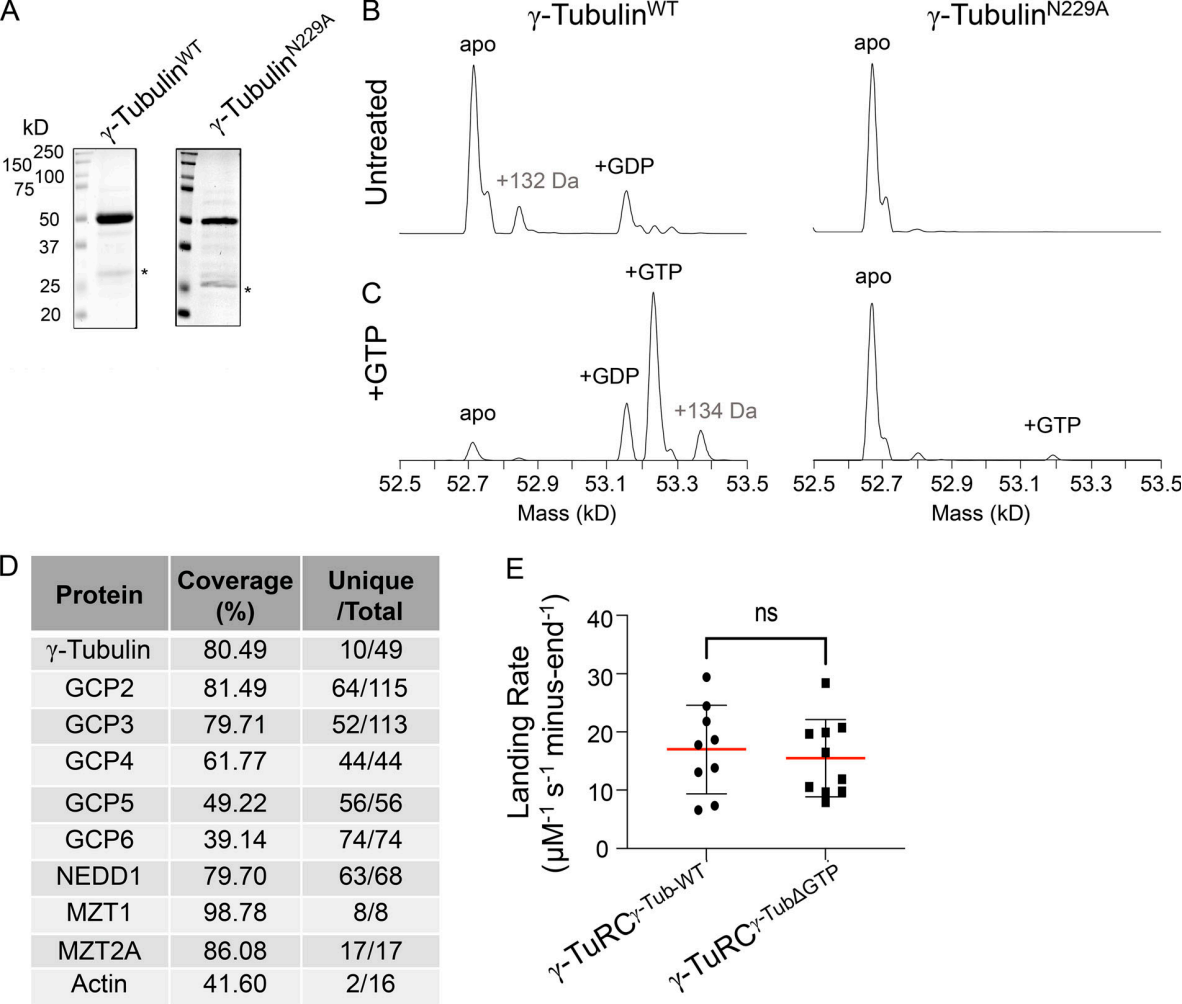
**Native mass spectrometry analysis of γ-tubulin**^N229A^ **and further analysis of γ-TuRC**^**γ-TubΔGTP**^**. (A)** SDS-PAGE analysis (Coomasie) of recombinant, purified γ-tubulin^WT^ (left) and γ-tubulin^N229A^ (right) after gel filtration. Asterisk indicates a contaminant at ∼25 kD. **(B and C)** Native mass spectrometry analysis of γ-tubulin^WT^ and γ-tubulin^N229A^ before (B) and after (C) incubation with MgGTP. **(D)** γ-TuRC proteins identified in liquid chromatography–mass spectrometry analysis of the γ-TuRC^γ-TubΔGTP^ complex. Coverage represents the percentage of identified protein sequences. “Unique/Total” designates the ratio of unique and total peptides identified. **(E)** Landing rates = number of capping events/[(µM γ-TuRC)×(experiment duration in seconds)×(number of total minus-ends)] of γ-TuRC^γ-Tub-WT^ or γ-TuRC^γ-TubΔGTP^ under short (10 min) duration experimental conditions. Mean (red line) and error (SD) are shown. γ-TuRC^γ-Tub-WT^, *n* = 9 replicates, γ-TuRC^γ-TubΔGTP^, *n* = 10 replicates, from *N* = 3 independent experiments. ns = not significant, unpaired two-sided Student’s t test, P = 0.65. Source data are available for this figure: SourceData FS1.

Next, we examined the role of GTP-binding to γ-tubulin within the context of the γ-TuRC holocomplex. To this end, we purified recombinant γ-TuRC^γ-TubΔGTP^, a complex that incorporates γ-tubulin with an N229A point mutation. While the homologous mutation has been shown to reduce GTP binding to yeast γ-tubulin (Gombos et al., 2013), its effect on human γ-tubulin has not yet been characterized. To analyze this, we also expressed and purified recombinant WT and N229A-γ-tubulin (Fig. S1 A). Native mass spectrometry indicated that WT γ-tubulin could bind nucleotide (Fig. S1, B and C). By contrast, GTP binding to N229A-γ-tubulin was suppressed (Fig. S1, B and C).

**Figure 2.**
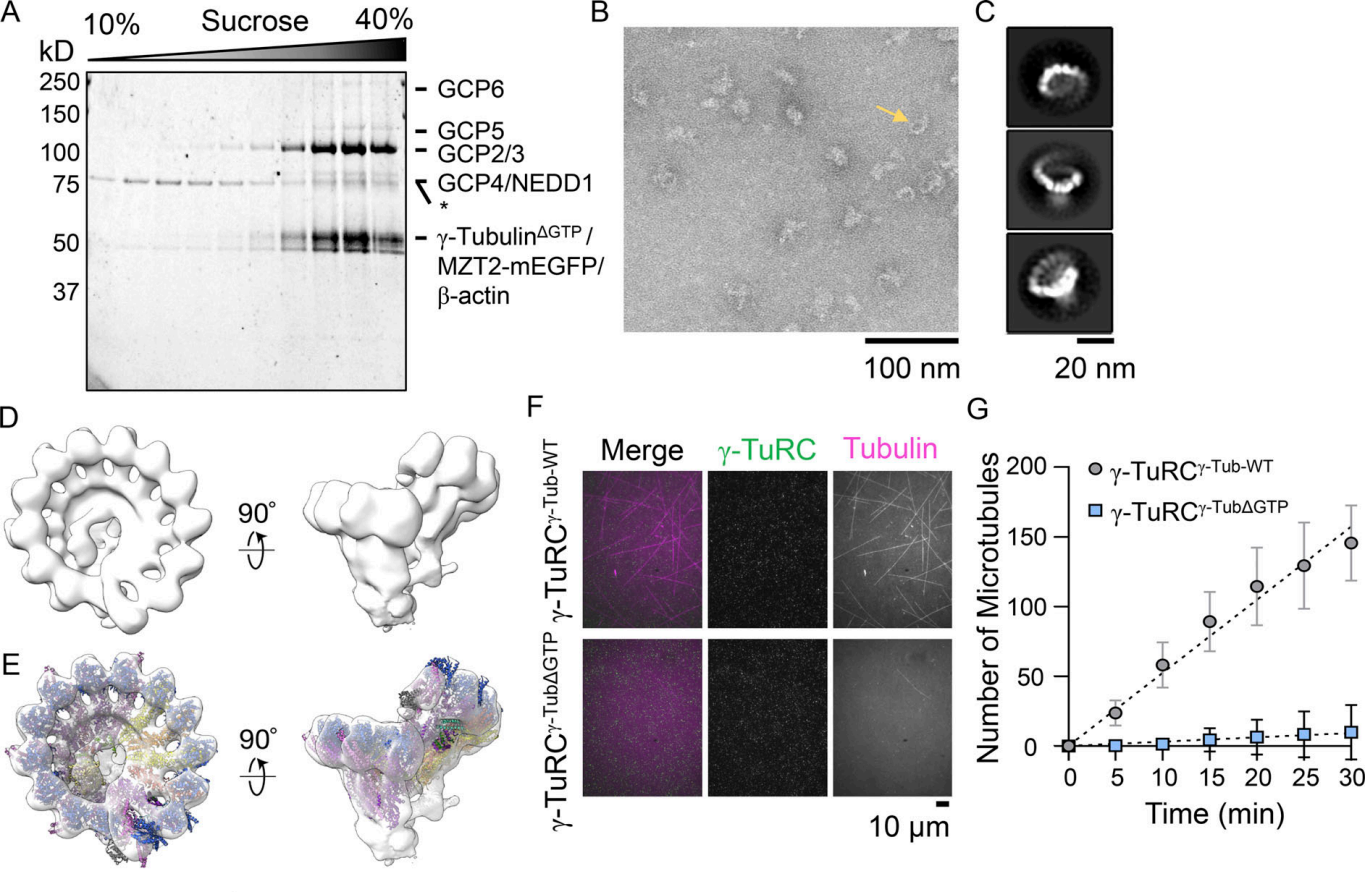
**Recombinant γ-TuRC**^**γ-TubΔGTP**^ **assembles into a 14-spoke assembly and cannot nucleate microtubules. (A)** SDS-PAGE analysis (Coomasie) of γ-TuRC^γ-TubΔGTP^ after sucrose gradient centrifugation and fractionation. The percentage (W/V) of sucrose is indicated at the top. Asterisk (*) indicates a 70-kD contaminant with a sedimentation peak at a lower sucrose percentage than the γ-TuRC^γ-TubΔGTP^ components. **(B)** Transmission EM micrograph of negatively stained γ-TuRC^γ-TubΔGTP^. Scale bar = 100 nm. **(C)** 2D averages showing three orientations of γ-TuRC^γ-TubΔGTP^ particles. Scale bar = 20 nm. **(D)** Two views of a 3D reconstruction of γ-TuRC^γ-TubΔGTP^. **(E)** Rigid body fit of the native human γ-TuRC model in the γ-TuRC^γ-TubΔGTP^ density map (Protein Data Bank accession nos. 6V6S, 6X0U, and 6X0V). **(F)** Images of nucleation assays in the presence of γ-TuRC^γ-Tub-WT^ (top) or γ-TuRC^γ-TubΔGTP^ (bottom). Two-color overlay of tubulin (magenta) and γ-TuRC^γ-TubΔGTP^ (green), and single-channel images are shown. Scale bar = 10 μm. **(G)** Quantification of the number of microtubules at the indicated time points per field of view for γ-TuRC^γ-Tub-WT^ or γ-TuRC^γ-TubΔGTP^ microtubule nucleation assays. Mean (symbols) and error (SD) are shown. Data were fitted using linear regression (dashed lines). *n* = 4 total replicates from *N* = 2 independent experiments. Source data are available for this figure: SourceData F2.

We characterized the γ-TuRC^γ-TubΔGTP^ in four ways. First, sucrose gradient centrifugation indicated that the complex incorporating mutant γ-tubulin migrates to ∼35% sucrose, a percentage comparable to what we have observed for recombinant WT and native complexes (Fig. 2 A; Wieczorek et al., 2020a; Wieczorek et al., 2021). Second, mass spectrometry analysis confirmed the presence of all 10 overexpressed γ-TuRC proteins (Fig. S1 D). Third, we used negative stain electron microscopy to characterize the overall structure of the complex. The γ-TuRC^γ-TubΔGTP^ appeared as asymmetric cones in negative-stain EM micrographs (Fig. 2 B). Reference-free 2D classification provided a dataset with different views of the complex, including some where individual spokes were discernible (Fig. 2 C). Particles corresponding to these 2D classes were then used to produce a 3D reconstruction of the complex, which revealed 14-spoke γ-TuRCs with a “seam” between the first and last spokes and a density in the lumen of the cone (Fig. 2 D). This low-resolution structure is consistent with recently published high-resolution cryo-EM structures of the human and *Xenopus* γ-TuRCs (Liu et al., 2020; Consolati et al., 2020; Wieczorek et al., 2020a). Correspondingly, a model of the native human γ-TuRC could be rigid body–fitted into the γ-TuRC^γ-TubΔGTP^ density (Protein Data Bank accession nos. 6V6S, 6X0U, and 6X0V; Wieczorek et al., 2020b; Wieczorek et al., 2020a; Fig. 2 E). Fourth, we examined the microtubule nucleation activity of the γ-TuRC^γ-TubΔGTP^ and found that microtubules were rarely observed in the γ-TuRC^γ-TubΔGTP^ sample in the course of the experiment (30 min). The few microtubules observed did not originate from GFP puncta. By contrast, the γ-TuRC^γ-Tub-WT^ sample revealed several GFP-puncta-associated microtubules within minutes (Fig. 2, F and G). Together, these data indicate that the N229A mutation in γ-tubulin suppresses the microtubule nucleation activity of the γ-TuRC holocomplex but does not substantially alter the overall structural organization of the complex. Additional studies will be needed to determine the high-resolution structure of the γ-TuRC^γ-TubΔGTP^ and analyze why this complex cannot nucleate microtubules.

### γ-TuRC^γ-TubΔGTP^ caps stable and dynamic microtubule minus-ends

**Figure 3.**
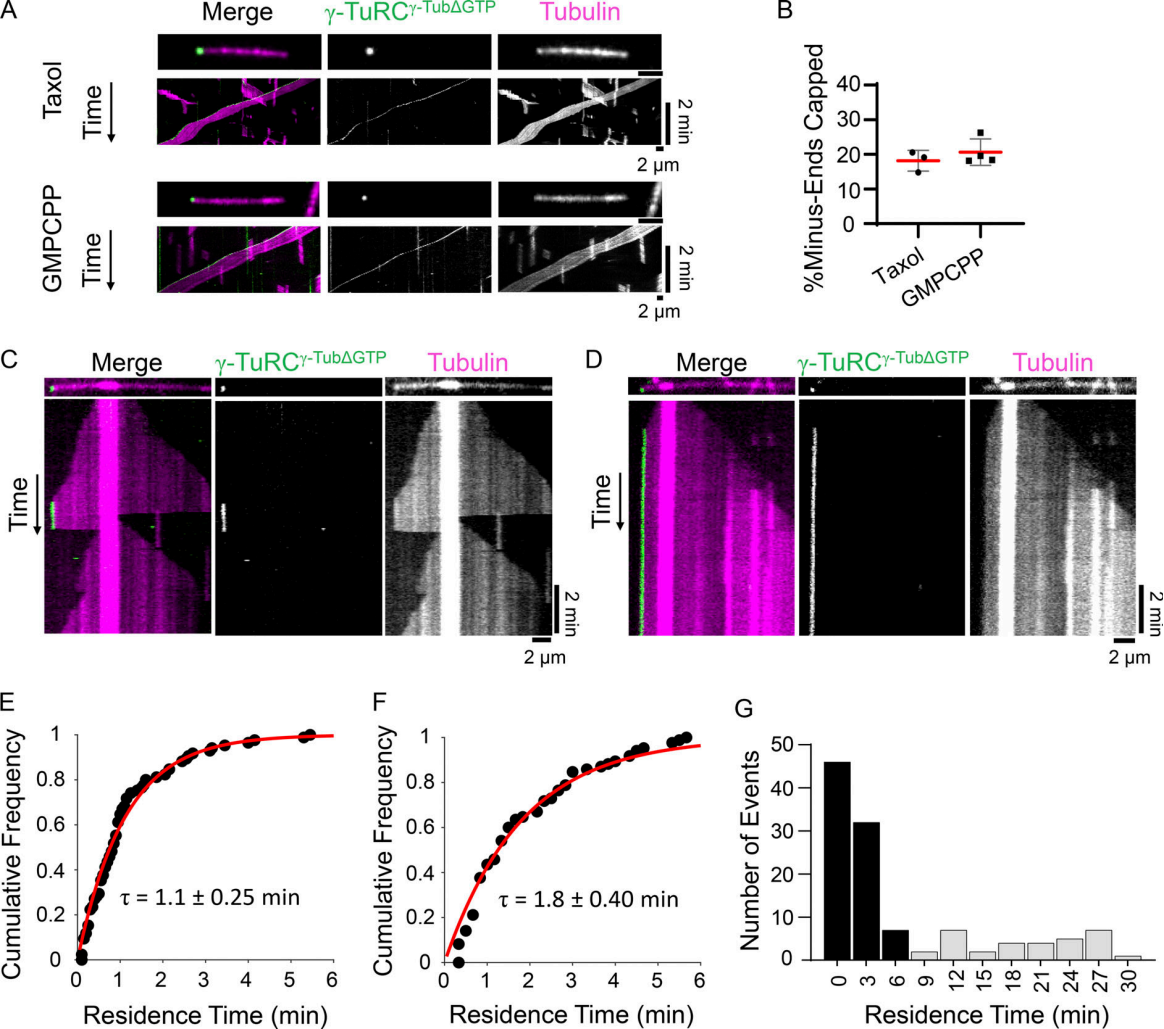
**Recombinant γ-TuRC**^**γ-TubΔGTP**^ **caps dynamic and stable microtubule minus-ends. (A)** Images and kymographs of γ-TuRC^γ-TubΔGTP^ capping taxol- or GMPCPP- stabilized microtubules bound to surface-immobilized kinesin motor domains. Two-color overlay of tubulin (magenta) and γ-TuRC^γ-Tub–WT^ (green), and single-channel images are shown. The images and kymographs are shown at different scales. **(B)** Quantification of the percentage of taxol- or GMPCPP-stabilized microtubule minus-ends capped by γ-TuRC^γ-TubΔGTP^ at 3 min from the start of imaging. Mean (red line) and error (SD) are shown. Taxol: *n* = 1,503 total microtubules from *N* = 3 independent experiments. GMPCPP: *n* = 1,634 total microtubules from *N* = 4 independent experiments. **(C and D)** Images and kymographs of γ-TuRC^γ-TubΔGTP^ capping events on dynamic microtubules. Two-color overlay of tubulin (magenta) and γ-TuRC^γ-TubΔGTP^ (green), and single-channel images are shown. **(E and F)** Cumulative frequency of the residence times of γ-TuRC^γ-TubΔGTP^ capping events where association and dissociation of the cap were observed under short (10 min; E) or long (30 min; F) duration experiments fitted to a single exponential (red line) with indicated mean residence time, τ. Error = 95% C.I. 10 min: *n* = 85 total events (81% of total) from *N* = 3 independent experiments. 30 min: *n* = 85 events (77% of total) from *N* = 2 independent experiments. **(G)** Frequency distribution of γ-TuRC^γ-Tub-WT^ residence times from longer duration experiments (30 min). Events where γ-TuRC^γ-TubΔGTP^ dissociation is observed (black bars) and where γ-TuRC^γ-TubΔGTP^'s minus-end association persisted (gray bars) are plotted. Bin size = 3 min. *n* = 111 total events from *N* = 2 independent experiments. Scale bars: distance (horizontal) = 2 µm, time (vertical) = 2 min.

We next examined microtubule capping by γ-TuRC^γ-TubΔGTP^ and first focused on stabilized microtubules. Under assay conditions similar to those used to examine the γ-TuRC^γ-Tub-WT^ (Fig. 1 D), puncta of γ-TuRC^γ-TubΔGTP^ (50 pM) were observed to bind the minus-ends of taxol- and GMPCPP-stabilized microtubules (Fig. 3 A). The percentage of capped minus-ends was 18 ± 3% (*n* = 1,503 microtubules from *N* = 3 independent experiments) for taxol-stabilized microtubules and 21 ± 4% (*n* = 1,634 microtubules from *N* = 4 independent experiments) for GMPCPP-stabilized microtubules (Fig. 3, A and B). Qualitatively, the γ-TuRC^γ-TubΔGTP^ cap at most stable microtubule minus-ends lasted for at least 2 min, as observed for γ-TuRC^γ-Tub-WT^ in this assay.

We next analyzed the capping of dynamic microtubules by γ-TuRC^γ-TubΔGTP^. Puncta of γ-TuRC^γ-TubΔGTP^ (10–30 pM) were observed to bind the minus-ends of dynamic microtubules (Fig. 3, C and D). The capping activity of the γ-TuRC^γ-TubΔGTP^ was qualitatively similar to that of the γ-TuRC^γ-Tub-WT^. The binding of γ-TuRC^γ-TubΔGTP^ suppressed microtubule dynamics at minus-ends (Fig. 3, C and D), and minus-end growth/shrinkage resumed following γ-TuRC^γ-TubΔGTP^ dissociation (Fig. 3 C). Notably, we observed that in the majority of events (∼81%, *n* = 105 total events from *N* = 3 independent experiments), the γ-TuRC^γ-TubΔGTP^ associated with and then dissociated from the minus-end, similar to the experiments performed with γ-TuRC^γ-Tub-WT^. The cumulative frequency distribution of these events provided a mean residence time of 1.1 ± 0.25 min (95% C.I.; time interval: 3 s, total time: 10 min; Fig. 3 E). The remaining events (∼19%) did not show γ-TuRC^γ-TubΔGTP^ dissociation throughout the course of the experiment (10 min). Longer acquisitioning duration conditions (frame interval: 10 s, total time: 30 min) showed a similar distribution of events. The majority of events (∼77%) showed both binding and dissociation of the γ-TuRC^γ-TubΔGTP^ with a mean residence time of 1.80 ± 0.40 min (95% C.I., Fig. 3, F and G, black bars), while a smaller percentage of events (∼23%, *n* = 111 total events from *N* = 2 independent experiments) did not show the dissociation of the γ-TuRC^γ-TubΔGTP^ (Fig. 3 G, gray bars).

We further compared the γ-TuRC^γ-TubΔGTP^ and γ-TuRC^γ-Tub-WT^ capping activities by measuring the landing rate, referring to the number of capping events divided by the concentration of the γ-TuRC, the time duration of the experiment, and the number of dynamic microtubules. For the γ-TuRC^γ-Tub-WT^, the average landing rate was 17.5 ± 7.8 µM^−1^ sec^−1^ minus-end^−1^ (*n* = 9 replicates from *N* = 3 independent experiments, Fig. S1 E). The average landing rate for the γ-TuRC^γ-TubΔGTP^ was 15.9 ± 7.0 µM^−1^ sec^−1^ minus-end^−1^ (*n* = 10 replicates from *N* = 3 independent experiments, Fig. S1 E), which was not significantly different from the γ-TuRC^γ-Tub-WT^ landing rate (unpaired two-tailed Student’s *t* test, P = 0.65).

Overall, these data suggest that the capping of stabilized and dynamic microtubule ends by γ-TuRC does not depend on GTP binding by γ-tubulin.

### Non-centrosomal microtubules form in the presence of γ-tubulin^ΔGTP^

**Figure 4.**
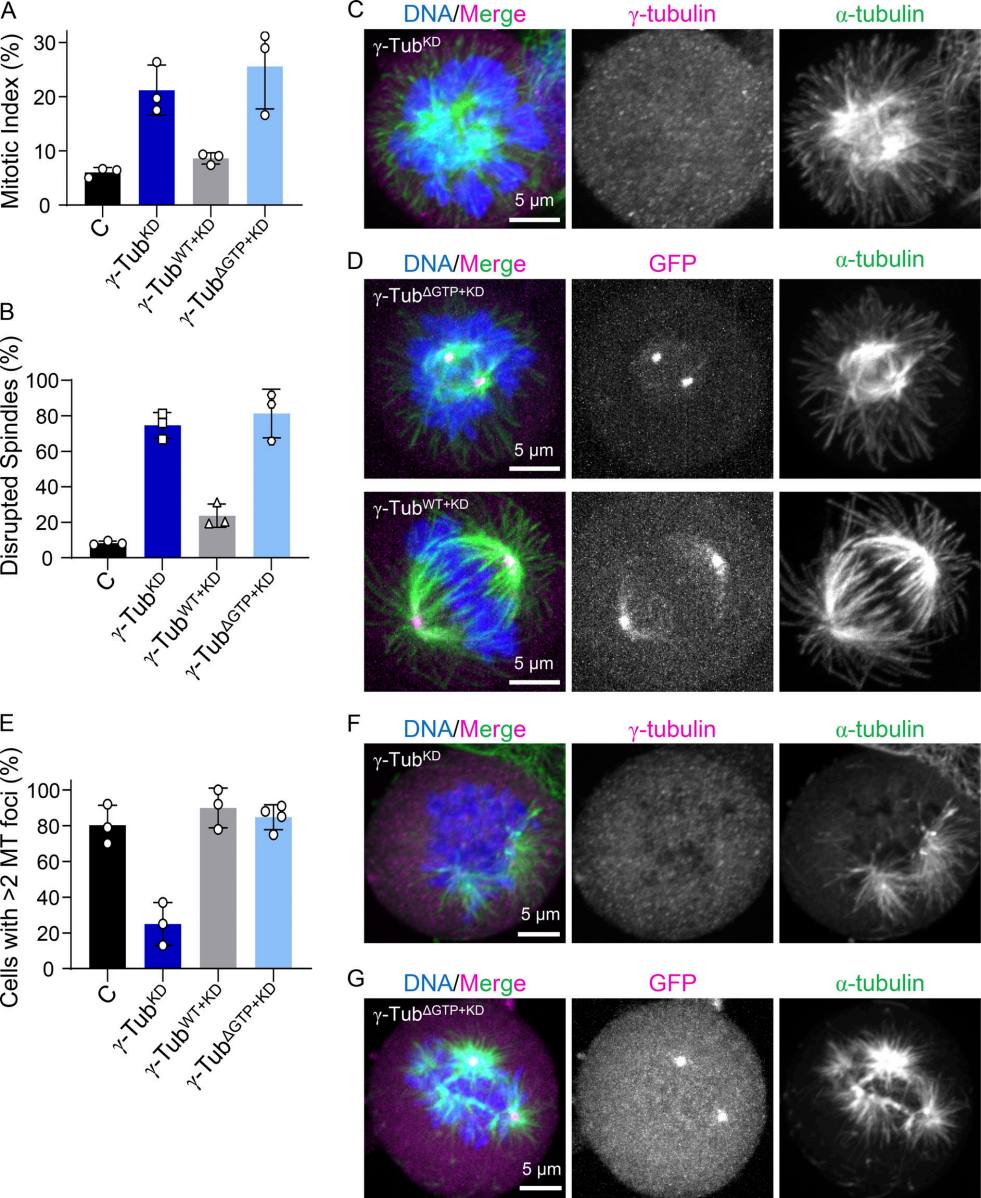
**γ-tubulin**^**ΔGTP+KD**^ **cells form disrupted spindles but display non-centrosomal microtubule formation. (A)** Analysis of the mean mitotic index. *n* = >2,000 cells per condition from *N* = 3 independent experiments. **(B)** The mean percentage of mitotic cells that displayed disrupted spindles. *n* = >200 cells per condition from *N* = 3 independent experiments. **(C and D)** Images of fixed mitotic γ-tubulin^KD^, γ-tubulin^ΔGTP+KD^, and γ-tubulin^WT+KD^ cells. Single-channel images (maximum-intensity projections) and overlays show γ-tubulin (immunofluorescence; C) or GFP (fluorescent signal; D; magenta), α-tubulin (green), and DNA (blue). **(E)** Quantification of the mean percentage of mitotic cells that display >2 microtubule foci at 5 min after nocodazole washout. *n* = >45 cells per condition from *N* ≥ 3 independent experiments. **(F and G)** Images of fixed mitotic γ-tubulin^KD^ and γ-tubulin^ΔGTP+KD^ cells 5 min after nocodazole washout. Single-channel images (maximum-intensity projections) and overlays show γ-tubulin (immunofluorescence; C) or GFP (fluorescent signal; D; magenta), α-tubulin (green), and DNA (blue). Scale bars = 5 µm. Error bars = standard deviation (A, B, and E).

**Figure S2.**
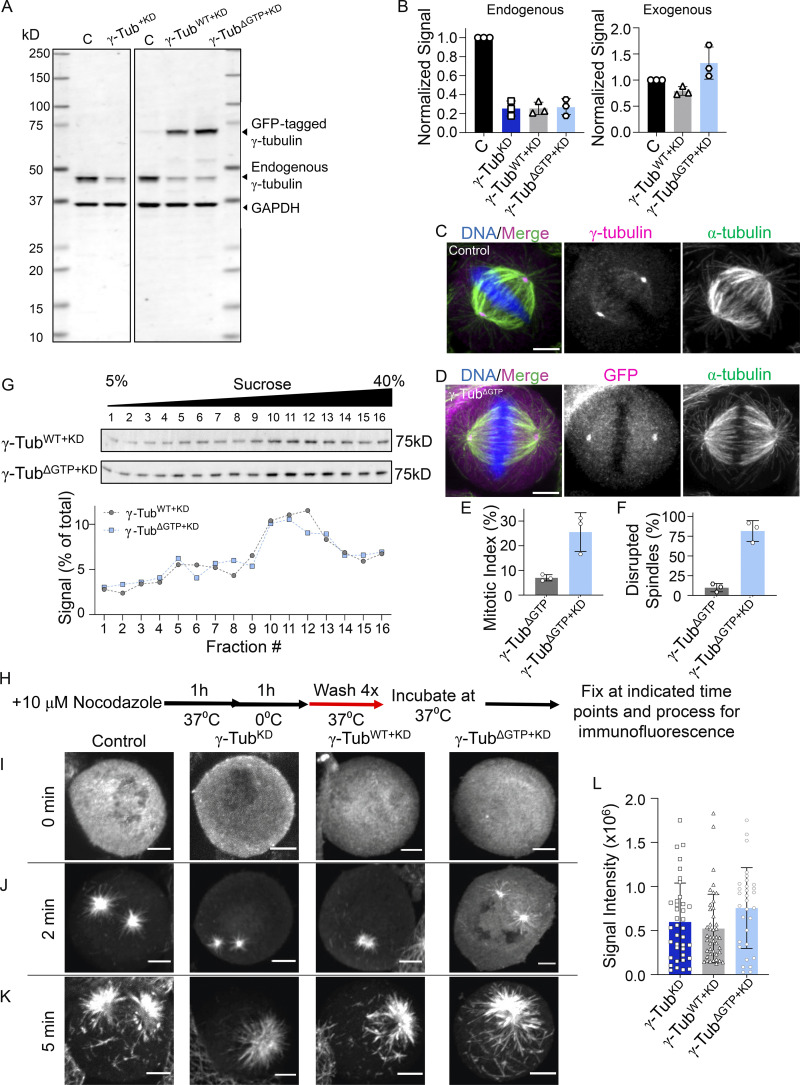
**Characterization of γ-tubulin cell lines and microtubule regrowth assay in fixed cells. (A)** Western blot analysis of cell lysates. Uncropped blots are shown. Bands corresponding to the expected molecular weights of GFP-tagged γ-tubulin, endogenous γ-tubulin, and GAPDH are indicated, along with the corresponding molecular weight standard. **(B)** Quantification of endogenously and exogenously expressed γ-tubulin levels in these cell lines determined by Western blotting, relative to loading control (GAPDH). The signal relative to the control is plotted. Mean and error (SD) are shown. *N* = 3 independent experiments. **(C and D)** Images of fixed, untransfected control (C) and γ-tubulin^ΔGTP^ (D) cells. Single-channel images (maximum-intensity projections) and overlays show (C) γ-tubulin (immunofluorescence, magenta) or (D) GFP (fluorescent signal; magenta), α-tubulin (green), and DNA (blue). **(E)** Quantification of the mean mitotic index in γ-tubulin^ΔGTP^ and γ-tubulin^ΔGTP+KD^ cells (values from Fig. 3 A are shown for comparison). *n* = >2,000 cells counted per condition from *N* = 3 independent experiments. **(F)** Mean percentage of mitotic cells that display disrupted spindles in γ-tubulin^ΔGTP^ and γ-tubulin^ΔGTP+KD^ cells (values from Fig. 3 B are shown for comparison). *n* = >200 cells counted per condition from *N* = 3 independent experiments. **(G)** Analysis of whole-cell lysates from γ-tubulin^WT+KD^ and γ-tubulin^ΔGTP+KD^ cell lines using sucrose gradient centrifugation. Western blot of γ-tubulin (top) and quantification of the percentage of γ-tubulin antibody signal within each sucrose gradient fraction (bottom) are shown. **(H)** Schematic of fixed cell microtubule regrowth assay. Cells were fixed at the indicated time points and processed for immunofluorescence. **(I–K)** Images of fixed mitotic cells at 0 (I), 2 (J), and 5 (K) min after nocodazole washout. Single-channel images (maximum-intensity projections) of α-tubulin are shown. **(L)** Quantification of the mean microtubules fluorescent signal at 2 min after nocodazole washout in the indicated cell lines. *n* = >30 cells per condition from *N* = 3 independent experiments. Scale bars = 5 µm. Error bars = SD (B, E, F, L). Source data are available for this figure: SourceData FS2.

While γ-tubulin depletion results in mild phenotypes during interphase, γ-tubulin has been shown to play a key role in regulating mitotic progression and spindle assembly (Strome et al., 2001; Hannak et al., 2002; Lüders et al., 2006; Hutchins et al., 2010; Tsuchiya and Goshima, 2021). Yet, it is unknown if the γ-TuRC’s capping activity contributes to these processes. Therefore, we next examined the role of nucleotide-binding deficient γ-tubulin in dividing cells. We first generated a cell line with an inducible expression of shRNA to γ-tubulin (hereafter, γ-tubulin^KD^). Relative to untransfected control cells (mitotic index:  6 ± 1%, *n* = 3,653 total cells, from *N* = 3 independent experiments), γ-tubulin knockdown resulted in an increase in the mitotic index (21 ± 5%, *n* = 4,800 total cells from *N* = 3 independent experiments) and an increase in cells displaying misaligned chromosomes and poorly separated spindle poles (hereafter, disrupted spindles; 75 ± 7%, *n* = 1,009 total cells from *N* = 3 independent experiments; control cells: 8 ± 1%, *n* = 205 total cells, from *N* = 3 independent experiments; Fig. 4, A–C). These phenotypes are consistent with prior work (Haren et al., 2006; Choi et al., 2010). While Western blot analysis showed that residual amounts of endogenous γ-tubulin remained in whole cell lysates (∼25%, Fig. S2, A and B), γ-tubulin puncta were not observed by immufluorescence, consistent with the loss of γ-tubulin in mitotic cells with disrupted spindles (Fig. 4 C and Fig. S2 C).

We next used the γ-tubulin^KD^ cell line to generate “addback” cell lines, which upon treatment with doxycycline, expressed both shRNA and RNAi-resistant C-terminally GFP-tagged WT or GTP-binding deficient γ-tubulin-N229A (hereafter, γ-tubulin^WT+KD^ and γ-tubulin^ΔGTP+KD^). In these cell lines, the levels of γ-tubulin^WT^–GFP and γ-tubulin^ΔGTP^–GFP were similar to levels of endogenous γ-tubulin in control cells, and the knockdown efficiencies were comparable with that of γ-tubulin^KD^ (Fig. S2, A and B). Importantly, γ-tubulin^ΔGTP+KD^ cells displayed a ∼fourfold increase in the mitotic index (26 ± 8%, *n* = 2,801 total cells from *N* = 3 independent experiments), relative to untreated controls, and an increase in the fraction of cells with disrupted spindles (81 ± 14%, *n* = 741 total cells from *N* = 3 independent experiments; Fig. 4, A and B). In contrast, the γ-tubulin^WT+KD^ cells had a mitotic index (9 ± 1%, *n* = 2,923 total cells from *N* = 3 independent experiments) and fractions of disrupted spindles (24 ± 7%, *n* = 253 from *N* = 3 independent experiments) comparable with untransfected controls (Fig. 4, A and B). These phenotypes were dependent on the depletion of endogenous γ-tubulin since a cell line expressing γ-tubulin^ΔGTP^–GFP, but not the shRNA, did not display mitotic defects (mitotic index = 7 ± 1%, *n* = 2,457 total cells and disrupted spindles = 10 ± 5%, *n* = 187 total cells from *N* = 3 independent experiments; Fig. S2, D–F). Importantly, both γ-tubulin^ΔGTP^–GFP and γ-tubulin^WT^–GFP localized to centrosomes (Fig. 4 D), and sucrose gradients of cell lysates showed a peak for γ-tubulin at ∼30% sucrose (Fig. S2 G), consistent with the incorporation of γ-tubulin^ΔGTP^–GFP and γ-tubulin^WT^–GFP into γ-TuRC complexes (Haren et al., 2020; Tsuchiya and Goshima, 2021).

We next performed a microtubule regrowth assay to examine how nucleotide-binding-deficient γ-tubulin affected cellular microtubule formation (Fig. S2 H). In different cell lines, at the earliest time points (2 min), microtubules grew predominantly from two sites with similar fluorescence intensities, consistent with growth from centrosomes (Fig. S2 I–L). At later time points (5 min), several additional microtubule foci were observed in control and γ-tubulin^WT+KD^ cells (80 ± 11% and 90 ± 11% of cells, *n* = 67 and 50 total cells from *N* = 3 independent experiments, respectively; Fig. S2 K). In the case of the γ-tubulin^KD^ cells, few additional microtubule foci formed at later time points (5 min; 27 ± 12% of cells; *n* = 79 total cells from *N* = 3 independent experiments, Fig. 4, E and F; Fig. S2, I–K). These findings are consistent with previous studies (Lüders et al., 2006; Cota et al., 2017). Importantly, γ-tubulin^ΔGTP+KD^ cells showed several foci at non-centrosomal sites at these later time points (88 ± 4%, *n* = 95 total cells from *N* = 4 independent experiments; Fig. 4, E and G; Fig S2, I–K). Together, these data suggest that addback of γ-tubulin^ΔGTP^–GFP allows for microtubule formation at non-centrosomal sites.

### Microtubule regrowth assays in live cells show non-centrosomal microtubule foci formation and coalescence

**Figure S3.**
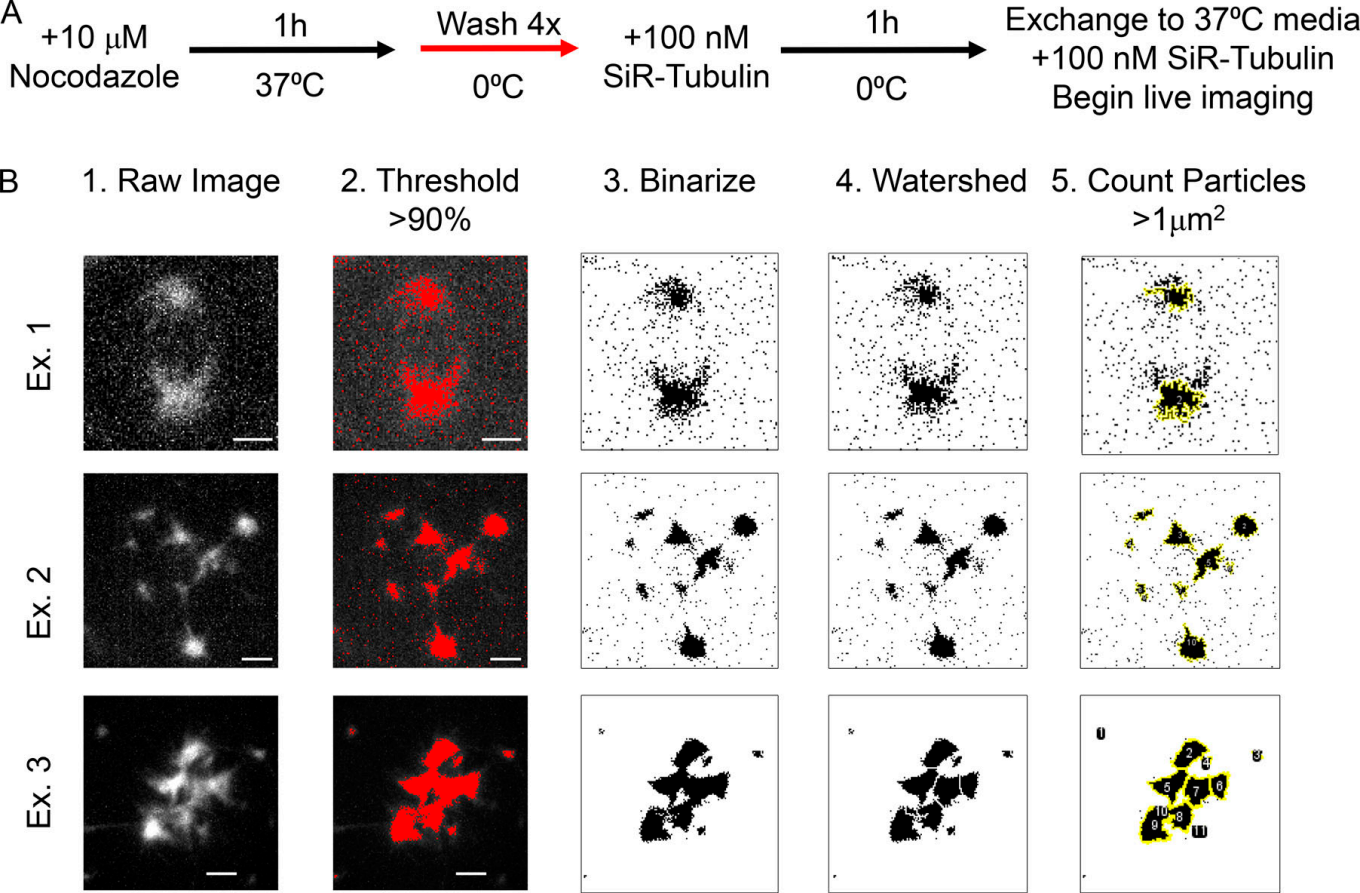
**Analysis of live cell microtubule regrowth assays. (A)** Schematic of the live cell microtubule regrowth assay. **(B)** Examples demonstrating the workflow for counting the number of microtubule foci. (1) The raw images were compiled as maximum intensity projections. (2) A signal intensity threshold of >90% was applied. (3) The image was binarized. (4) The signal was segmented using the Watershed plugin in FIJI. (5) Any particles greater than 1 µm^2^ were counted using the Analyze Particles tool in FIJI. Example 1 illustrates this workflow in a γ-tubulin^KD^ cell. Examples 2 and 3 illustrate this workflow in γ-tubulin^WT+KD^ and γ-tubulin^ΔGTP+KD^ cells where the foci were either small and dispersed (Example 2), or large and clustered together (Example 3). Scale bar = 2.5 µm.

We next repeated the microtubule regrowth assay in live cells to further characterize the course of microtubule foci formation and to exclude the possibility that the multiple sites of microtubule growth stemmed from the fragmentation of one or two sites of growth (Fig. S3 A). To avoid further modification of the γ-tubulin cell lines, we utilized the microtubule stain SiR–tubulin (Lukinavičius et al., 2014). We found that microtubule foci became visible at 5–10 min following the exchange to warm media without nocodazole (Fig. S3 A). This delay relative to the fixed cell assay may be, in part, due to dim SiR–tubulin labeling of newly formed microtubules (David et al., 2019). Additionally, Z-stacks were acquired as the microtubule foci were dynamic within the cytoplasm. The number of Z-planes was limited to minimize phototoxicity; consequently, one of the two centrosomes, identified as bright GFP puncta in cells expressing GFP-tagged γ-tubulin, was sometimes outside of the Z-planes acquired at certain time points.

**Figure 5.**
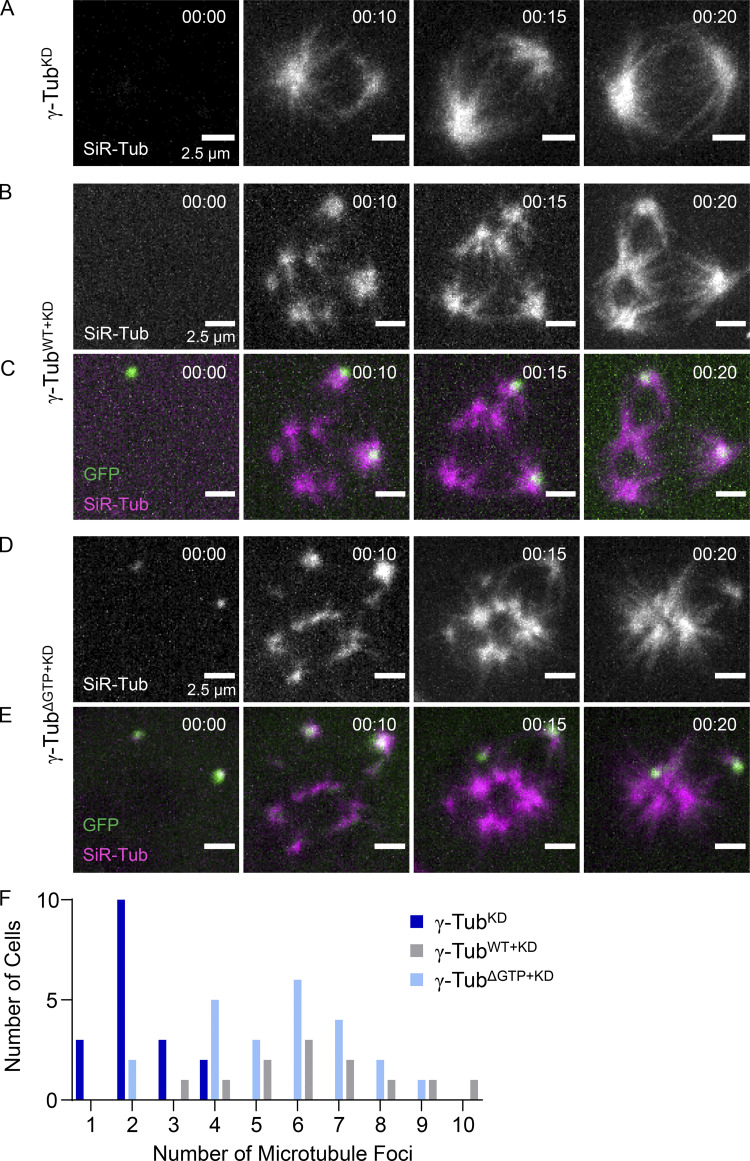
**Microtubule foci formation and coalescence in live γ-tubulin**^**ΔGTP+KD**^ **cells. (A–E)** Live imaging of microtubule regrowth assay by spinning disc confocal microscopy in γ-tubulin^KD^ (A; no GFP signal), γ-tubulin^WT+KD^ (B and C), and γ-tubulin^ΔGTP+KD^ (D and E) cells. Maximum-intensity projections at individual time points are shown. Timestamps = hh:mm. Single-channel images (A, B, D) show SiR–tubulin-labeled microtubules. Overlays (C and E) show GFP-tagged γ-tubulin (green) and SiR–Tubulin-labeled microtubules (magenta). Scale bar = 2.5 µm. **(F)** Quantification of the number of microtubule foci in γ-tubulin^KD^ (dark blue bars, *n* = 18 total cells from *N* = 2 independent experiments), γ-tubulin^WT+KD^ (gray bars, *n* = 13 total cells from *N* = 3 independent experiments), and γ-tubulin^ΔGTP+KD^ (light blue bars, *n* = 23 total cells from *N* = 3 independent experiments) cells.

In γ-tubulin^KD^ cells, SiR–tubulin-labeled microtubules appeared at predominantly two foci, consistent with our fixed-cell experiments (Fig. 4 F and Fig. 5 A). In contrast, cells expressing γ-tubulin^WT^–GFP displayed several microtubule foci throughout the cytoplasm (Fig. 5, B and C). Non-centrosomal sites could be discerned as the centrosomes were marked by bright GFP puncta (Fig. 5 C). As in our fixed cell analysis, γ-tubulin^ΔGTP+KD^ also displayed several microtubule foci at sites other than the centrosomes (Fig. 4 G and Fig. 5, D and E). In γ-tubulin^KD^ cells, the number of microtubule foci ranged from 1 to 4 at 10 min (total mean = 2 ± 1, *n* = 18 total cells from *N* = 2 independent experiments; Fig. 5 F and Fig. S3 B). In contrast, the number of foci in γ-tubulin^WT+KD^ cells ranged from 3 to 10 (total mean = 7 ± 2, *n* = 13 total cells from *N* = 3 independent experiments; Fig. 5 F and Fig. S3 B), and in γ-tubulin^ΔGTP+KD^ cells from 2 to 9 (total mean = 6 ± 2, *n* = 23 total cells from *N* = 3 independent experiments; Fig. 5 F and Fig. S3 B). In both γ-tubulin^WT+KD^ and γ-tubulin^ΔGTP+KD^ cells, centrosomal and non-centrosomal foci grew brighter as time progressed. As the signal intensity of these foci increased, they also began to coalesce (Fig. 5, B–E). Together, these data suggest that addback of nucleotide-binding deficient γ-tubulin, in dividing cells lacking endogenous γ-tubulin, leads to microtubule foci formation at non-centrosomal sites.

In summary, our studies characterize the γ-TuRC’s capping activity in biochemical assays and cellular contexts. In light of our in vitro findings that the γ-TuRC^γ-TubΔGTP^ does not nucleate microtubules but can cap microtubule minus-ends, we propose that nucleotide-binding deficient γ-tubulin promotes the formation of non-centrosomal microtubules in dividing cells through its capping activity. These microtubules are likely nucleated through pathways within the spindle or at kinetochores that may involve proteins such as TPX2 and CLASP1, which have been proposed to nucleate non-centrosomal microtubules during mitosis in the absence of γ-tubulin (Gruss et al., 2002; Tsuchiya and Goshima, 2021; Renda et al., 2022).

We suggest a model where γ-TuRC’s capping activity can suppress minus-end dynamics and also mediate microtubule transport and organization within the bipolar spindle. While it is not known how γ-TuRC capping regulates the lifetime of microtubules in dividing cells, we note that the turnover of individual spindle microtubules has been found to be on a similar 1-min time scale as our measured residence times for γ-TuRC capping of dynamic microtubules (Needleman et al., 2010). Additionally, fluorescently tagged γ-tubulin molecules have been found to interact transiently within the spindle and to traverse the length of the half spindle (5–10 µm) within ∼2 min (Hallen et al., 2008; Lecland and Lüders, 2014). Furthermore, the γ-TuRC has been shown to interact with dynein, a minus-end directed motor protein that contributes to the organization of microtubules at the spindle poles, whose motility in the spindle is on the order of 2–6 µm per minute (Heald et al., 1996; Merdes et al., 1996; Young et al., 2000; Lecland and Lüders, 2014). This model is consistent with our observation that the microtubule foci in γ-tubulin^ΔGTP+KD^ cells coalesce over time, which is likely to be a dynein-dependent process (Heald et al., 1996; Tulu et al., 2006).

Dissociation of the γ-TuRC from microtubule minus-ends would allow for the regulation of microtubule turnover, resumed minus-end dynamics, or the binding of other minus-end associated proteins (Martin and Akhmanova, 2018). Interestingly, we and others have found that centrosomal microtubule formation is not as severely affected by the loss of γ-TuRC components (Hannak et al., 2002; Lüders et al., 2006; Cota et al., 2017). This may be partially due to the localization of other minus-end capping proteins such as ASPM and NuMA at spindle poles or of centriolar and pericentriolar matrix proteins, which may form microtubules in a γ-TuRC-independent manner (Gaglio et al., 1995; Jiang et al., 2017; Woodruff et al., 2017; Baumgart et al., 2019; Watanabe et al., 2020).

Our findings that the lifetimes and nucleotide-dependencies differ between γ-TuRC capping of dynamic minus-ends and nucleation of new microtubules suggest that the type of contacts that are formed between γ-tubulin and α,β-tubulin are different during these types of events. As the reported structures of γ-tubulin do not reveal major conformational changes upon different nucleotide-bound states (Aldaz et al., 2005; Rice et al., 2008), it remains unclear how nucleotide-binding by γ-tubulin can impact γ-TuRC activities. Nonetheless, the regulation of γ-tubulin’s nucleotide-binding state may allow for modulation between long and short capping lifetimes. Interestingly, a recently-identified phosphorylation site within the nucleotide-binding pocket of yeast γ-tubulin likely interferes with nucleotide binding, suggesting a potential regulatory mechanism (Brilot et al., 2021). Together, our results suggest the role of the γ-TuRC’s capping activity in mitotic spindle assembly and may have implications for the formation and organization of microtubules in other contexts, such as in non-centrosomal microtubule organizing centers in differentiated cells (Sanchez and Feldman, 2017).

## Materials and methods

### Plasmids

The following plasmids were used in this study.

pACEBac1-γ-tubulin-TEV-HIS6 (Wieczorek et al., 2021).
pACEBac1-γ-tubulin^ΔGTP^-TEV-HIS6 (Wieczorek et al., 2021).
pACEBac1-γ-TuSC (Wieczorek et al., 2021).
pACEBac1-γ-TuRC-GFP (Wieczorek et al., 2021).
pACEBac1-γ-TuSC^γ-tubΔGTP^ (Wieczorek et al., 2021).
pACEBac1-γ-TuRC^γ-tubΔGTP^ was generated by replacing the wild-type γ-tubulin in pACEBac1-γ-TuRC-GFP with N229A-γ-tubulin.
pMal-C2-TEV protease (pRK793; Addgene plasmid 8827; Kapust et al., [2001]).
pET17b-K560 (gift from R.D. Vale, University of California, San Francisco, San Francisco, CA, USA, and Janelia Research Campus, Howard Hughes Medical Institute, Ashburn, VA, USA).

pSuperior.retro.puro-sh-γ-tubulin was generated with the target sequence 5′-A**G**GA**G**GAC​ATG​TT**C**AA**G**GA-3′ (Lüders et al., 2006; Choi et al., 2010) according to the manufacturer’s protocol (OligoEngine).

pCDNA5/FRT/TO–γ-tubulin^WT-RNAi-resistant^-GFP was generated by inserting the cDNA for γ-tubulin into the vector using restriction enzymes. Four silent mutations were introduced into the target sequence using site-directed mutagenesis by PCR (NCBI gene ID: 7283): G2228A, C2231T, C2237T, G2240A, noted as bold letters in the sh-RNA target sequence above. To account for any non-specific mutations that may have occurred in the vector backbone, the sequence-confirmed gene was excised by restriction enzymes and religated into the cut original vector.

pCDNA5/FRT/TO–γ-tubulin^ΔGTP-RNAi-resistant^-GFP was generated by site-directed mutagenesis of the WT plasmid. After confirmation of mutagenesis by sequencing, the gene was excised by restriction enzymes and religated into the cut original vector.

### Antibodies

The following primary and secondary antibodies were purchased from commercial sources, with their application and working concentrations indicated in parentheses.

Anti- γ-tubulin: mouse, Millipore Sigma, GTU-88 (Western blot: 1:000; immunofluorescence: 1:500). Anti-γ-tubulin: rabbit, Millipore Sigma, T5192 (immunofluorescence, 1: 250). Anti-GAPDH: mouse, ProteinTech, 1E6D9 (Western blot, 1:1,000). Anti-α-tubulin: rat, Invitrogen, clone YL1/2 (immunofluorescence: 1:500). FITC-conjugated Anti-β-tubulin: mouse, Sigma-Aldrich, F2043 (immunofluorescence, 1:1,000). Goat anti-Rat Alexa Fluor 594: Invitrogen, A-11007 (immunofluorescence, 1:1,000). Donkey anti-Rabbit Texas Red: Jackson Immunoresearch, 711-075-152 (immunofluorescence, 1:500). Goat anti-Mouse Alexa Fluor 488: Invitrogen, A-11001 (immunofluorescence, 1:500). Janelia Fluor 646-Hoechst: Janelia Materials (immunofluorescence, 1:1000). Goat polyclonal anti-Mouse IRDye 680RD: LI-COR, 926-68070 (Western blot, 1:10,000).

### Cell lines

HeLa TREx cells (Thermo Fisher Scientific) and Ampho293T (Thermo Fisher Scientific) were maintained in DMEM (Thermo Fisher Scientific) +10% FBS (Sigma-Aldrich). Cell lines containing tetracycline-inducible constructs were cultured in DMEM +10% Tet-System-Approved FBS (TakaraBio). Cells were incubated at 37°C and 5% CO_2_. Cells were used at early passage numbers (<20 passages). Cells were tested for mycoplasma using a PCR-based method (Uphoff and Drexler, 2014).

To generate HeLa cells with tetracycline-inducible control of γ-tubulin shRNA expression, we used retroviral transduction. First, retroviral particles containing the γ-tubulin shRNA were generated by transfecting Ampho293T cells via calcium phosphate with pSuperior.retro.puro-sh–γ-tubulin. After a 48-hr incubation, the media from the Ampho293T containing retroviral particles was harvested and filtered using a 0.45 µm filter, supplemented with 4 µg/ml polybrene, and then added to HeLa TREx cells. This process was repeated the following day, once in the morning and once in the evening. 48 h after the first application, stably transduced cells were selected with 2 µg/ml puromycin. Cells were maintained in puromycin until plating for the experiment. To induce shRNA expression, cells were allowed to adhere overnight and then treated for 72 h with 1 µg/ml doxycycline (Sigma-Aldrich).

To generate cells with inducible expression of shRNA-resistant γ-tubulin-GFP, we transfected both WT and ΔGTP pCDNA5/FRT/TO-γ-tubulin^RNAi-resistant^-GFP into HeLa TREx cells using Lipofectamine 2000 according to the manufacturer’s recommendations (Invitrogen). Hygromycin B (Invitrogen) was used to select cells that had stably incorporated the construct into the Flp-In site. Cells were maintained in Hygromycin B until plated for an experiment. This method was performed using non-transduced HeLa TREx cells or with HeLa TREx cells already transduced to stably incorporate inducible γ-tubulin shRNA. Expression of WT and ΔGTP γ-tubulin^RNAi-resistant^–GFP was induced by allowing the cells to adhere overnight and subsequently treating the cells with 1 µg/ml doxycycline for 48 h.

### Expression and purification of γ-TuRC^γ-tub-WT^ and γ-TuRC^γ-tubΔGTP^

γ-TuRC^γ-tub-WT^ and γ-TuRC^γ-tubΔGTP^ were purified using the same protocol, as described in Wieczorek et al. (2021). In brief, to purify γ-TuRC^γ-tubΔGTP^, pACEBac1-γ-TuSC^γ-tubΔGTP^ and pACEBac1-γ-TuRC^γ-tubΔGTP^ were each transfected into SF9 cells using Cellfectin transfection reagent according to the manufacturer’s protocol. Viruses from SF9 cells were amplified twice, after which they were used to infect Hi5 cells at a density of 3 × 10^6^ cells/ml.

After 60 h, the Hi5 cells were lysed in lysis buffer (40 mM Hepes, pH 7.5, 150 mM KCl, 1 mM MgCl2, 10% glycerol [vol/vol], 0.1% Tween-20, 0.1 mM MgATP, 0.1 mM MgGTP, 1 mM β-mercaptoethanol, complete EDTA-free Protease Inhibitor Cocktail tablets [Roche], 500 U benzonase, 2 mM PMSF, and 4 mM benzamidine-HCl) on ice using dounce homogenization. The lysate was clarified at 56,000 rpm in a Type 70Ti rotor for 1 h at 4°C. The clarified lysate was filtered and loaded onto a 1-ml NHS-Trap column preconjugated with rabbit IgG. The column was washed first with lysis buffer and then with gel filtration buffer (40 mM Hepes, pH 7.5, 150 mM KCl, 1 mM MgCl_2_, 10% glycerol [vol/vol], 0.1 mM MgGTP, and 1 mM β-mercaptoethanol). 1 mg of TEV protease, diluted in gel filtration buffer supplemented with 0.1 mM MgGTP, was loaded onto the column and incubated for 1 h at 4°C. The eluted fractions were pooled and dialyzed against dialysis buffer (40 mM Hepes, pH 7.5, 150 mM KCl, 1 mM MgCl_2_, 60% sucrose [wt/vol], 0.1 mM MgGTP, and 2 mM β-mercaptoethanol) for 4 h at 4°C or until the volume was reduced to <1 ml. The dialyzed eluate was then gel-filtered using a Superose 6 increase 10/300 column (Cytvia) preequilibrated in gel filtration buffer. The pACEBac1-γ-TuRC^γ-tubΔGTP^ was eluted at ∼9 ml.

The peak fraction was further purified by sucrose gradient centrifugation by loading the eluate on a 2 ml 10–40% sucrose (wt/vol) gradient. The sucrose for four gradient steps was dissolved in gradient buffer (40 mM HEPES, pH 7.5, 150 mM KCl, 1 mM MgCl_2_, 0.01% Tween-20 [vol/vol], 0.1 mM MgGTP, and 1 mM β-mercaptoethanol). The gradients were centrifuged for 3 h at 50,000 rpm at 4°C in a TLS-55 swing bucket rotor with minimum acceleration and no break on the centrifuge. 10, 200-µl fractions were collected from the top of the gradient manually using a cut P1000 tip. A sample from each was analyzed by SDS-PAGE followed by Coomasie staining. Peak fractions were aliquoted, snap-frozen, and stored in liquid N2 at −80°C. The concentration of the purified γ-TuRC was determined by quantitative Western blotting.

### γ-TuRC microtubule nucleation assay

TIRF-based microtubule nucleation assays using γ-TuRC^γ-tub-WT^ and γ-TuRC^γ-tubΔGTP^ were performed as described in Wieczorek et al. (2021). Assays using wild-type and mutant complexes were performed on the same days using the same reagents.

For data analysis, time-lapse images were drift-corrected using the Multi-StackReg plugin in Fiji (Thevenaz et al., 1998). To quantify the residence time of the γ-TuRC at minus-ends following a nucleation event, 15 µM tubulin was used in the reaction so that individual microtubules could be resolved over time. We generated kymographs for microtubules that were determined to have been nucleated from a surface-immobilized γ-TuRC, i.e., the initiation of microtubule growth off of a green γ-TuRC puncta was fully observed. A microtubule was considered dissociated from the γ-TuRC if the microtubule was released from the γ-TuRC or if the microtubule minus-end grew following nucleation by γ-TuRC. Spontaneously nucleated microtubules, identified as microtubules growing at both ends or microtubules that appeared in the TIRF field of view after the initial growth phase, were not included in our analyses.

To compare the nucleation efficiencies of the γ-TuRC^γ-tub-WT^ and γ-TuRC^γ-tubΔGTP^, 1pM of γ-TuRC adhered to the coverslip and 20 µM tubulin was introduced to the flow chamber. Microtubules observed within the FOV (132 × 132 µm^2^) at the indicated time points were counted manually and tracked using the Line tool together with the ROI manager tool in FIJI. The number of newly nucleated microtubules at each time point was added to the number of microtubules counted at the previous time point to report the total number of microtubules.

### Dynamic minus-end-capping TIRF assay

The TIRF microscope and flow cell setup were the same as described for the microtubule nucleation assay with the following modifications. The flow cell was rinsed with BRB80 (80 mM K-PIPES, pH 6.8, 1 mM MgCl_2_, and 1 mM EGTA) containing 1 mM TCEP, followed by 0.2 mg/ml PLL-PEG-biotin (PLL(20)-g[3.5]–PEG(2)/PEG(3.4)–biotin(20%); SuSos) prepared in BRB80 + 1 mM TCEP. After 5 min, the flow cell was rinsed with BRB80 + 1 mM TCEP, and a mixture containing 0.5 mg/ml k-casein and 0.25 mg/ml neutravidin prepared in BRB80 + 1 mM TCEP was flowed in. After 5 min, GMPCPP seeds containing 12.5% X-rhodamine-labeled tubulin and 1% biotinylated tubulin, prepared with two cycles of polymerization as described (Ti et al., 2016), were flowed in. After 5 min, unbound seeds were rinsed off with room-temperature assay buffer (BRB80 + 1 mM TCEP, 50 mM KCl, 0.2 mg/ml k-casein, and 1 mM MgGTP). A reaction mixture containing 10–30 pM γ-TuRC, 15 µM tubulin (containing ∼2.5% X-rhodamine-labeled tubulin), and oxygen scavengers (0.035 mg/ml catalase, 0.2 mg/ml glucose oxidase, 2.5 mM glucose, and 10 mM DTT) was prepared in assay buffer and introduced to the flow cell. Experiments with γ-TuRC^γ-tub-WT^ and γ-TuRC^γ-tubΔGTP^ were performed on the same days using the same reagents. Soluble tubulin was prepared by mixing unlabeled and rhodamine-labeled tubulin on ice and clarifying at 90,000 x g for 10 min at 4°C and held on ice for no more than 2 h, after which a new sample of soluble tubulin was prepared. The tubulin concentration was measured using a Bradford assay. The flow cell was sealed with VALAP and placed on the TIRF microscope stage. The sample was imaged immediately to observe microtubule growth at both ends so that the plus-ends and minus-ends were distinguishable. Images were acquired for either 10 min at 3-s intervals or 30 min at 10-s intervals and an exposure time of 500 ms for each laser channel. The microscope chamber was heated to ∼35–37°C before image acquisition. Image acquisition was controlled using NIS- Elements AR 4.60.00 (Nikon).

### Analysis of γ-TuRC capping at dynamic microtubule minus-ends

Time-lapse images were drift-corrected using the MultiStackReg Fiji plugin (Thevenaz et al., 1998). To quantify minus-end capping by γ-TuRCs, we generated kymographs for microtubules that met the following criteria: (1) microtubules were determined to not be associated in parallel with another filament (i.e., a bundle) and (2) both growing ends of the microtubule were within the FOV. Next, a capping event was scored if it met the following criteria: (1) puncta persisted for two consecutive frames or greater, (2) the puncta were not present at the interaction site before the polymerizing microtubule reached that site. Distributions of the capping lifetimes were generated by measuring the length of time these criteria were met by first plotting the Z-axis profile of the γ-TuRC signal in FIJI and then using the tDetector algorithm (Chen et al., 2014) with an alpha value of 0.999 to detect the onset and end of the capping event. Since the γ-TuRC signal varied during the capping event due to drift and movement of the microtubule end, consecutive steps that were above the baseline signal were counted together and visually confirmed for each independent event.

The landing rate was determined by counting the number of capping events within each experiment and dividing by the total imaging time in minutes, the γ-TuRC concentration, and the number of GMPCPP seeds with polymerizing extensions in the field of view. Statistical analysis was performed using a two-sided Student’s *t* test in GraphPad Prism. The data was determined to be normal using the D’Agostino and Pearson test that was performed using GraphPad Prism.

### Stable taxol or GMPCPP-microtubule minus-end-capping

Stable taxol microtubules were prepared by incubating 10 µM tubulin (with ∼5% X-rhodamine-labeled tubulin) in BRB80 + 1 mM MgGTP +1 mM DTT. This tubulin mixture was incubated at 37°C for 2 min, after which increasing concentrations of taxol were added as follows: 1 µM taxol in DMSO at 1/10th the volume from 7 min, 10 µM taxol in DMSO at 1/10th the volume for 7 min, and 100 µM taxol in DMSO at 1/10th the volume for 15 min. After the final incubation period, the mixture was diluted five times with taxol buffer (BRB80 containing 10 µM taxol, 1% final DMSO concentration). The microtubules were centrifuged at 13,200 rcf for 15 min at RT. The microtubule pellet was washed with 200 μl of taxol buffer and then resuspended in 100 μl of taxol buffer. Taxol-stabilized microtubules were prepared fresh on each day of use and stored at RT.

Stable GMPCPP microtubules were prepared by incubating 10 µM tubulin (with ∼5% X-rhodamine-labeled tubulin) in BRB80 + 1 mM GMPCPP +1 mM DTT (Jena Biosciences). The mixture was incubated on ice for 5 min followed by 37°C for 30 min. The microtubules were then pelleted at 90,000 rpm for 5 min at 37°C using a preheated TLA 120.2 rotor. The supernatant was removed and the microtubules were resuspended in BRB80 + 1 mM DTT. Following a 20-min incubation on ice, 1 mM GMPCPP was added to the depolymerized tubulin. The mixture was transferred to 37°C and incubated for 1 h. Microtubules were then centrifuged as before and the obtained pellet was washed with 200 μl BRB0 + 1 mM DTT, followed by resuspension in 50 μl. GMPCPP-stabilized microtubules were used over the course of 4 d and stored at RT.

The TIRF microscope and flow-cell setup were the same as described for the microtubule nucleation assay. All buffers were kept at RT. The flow cell was rinsed with BRB80 (80 mM K-PIPES, pH 6.8, 1 mM MgCl_2_, and 1 mM EGTA) containing 1 mM TCEP followed by 700 nM of K560 and purified as described in Romberg et al. (1998). After 5 min, the flow cell was washed with assay buffer (50 mM KCl, 0.2 mg/ml k-casein, 1 mM MgGTP, 1 mM TCEP in BRB80). Next, 0.2 mg/ml PLL–PEG–biotin (PLL(20)-g[3.5]–PEG(2)/PEG(3.4)–biotin(20%); SuSos) + 0.5 mg/ml k-casein prepared in assay buffer were flowed in. After 5 min, taxol- or GMPCPP-stabilized microtubules diluted ∼1:50 in assay buffer were flowed in. After 5 min, unbound microtubules were rinsed off with a warm assay buffer. A reaction mixture containing 50 pM γ-TuRC and oxygen scavengers (0.035 mg/ml catalase, 0.2 mg/ml glucose oxidase, 2.5 mM glucose, and 10 mM DTT) was prepared in an assay buffer supplemented with 100 µM MgATP and introduced to the flow cell. For taxol-stabilized microtubules, all buffers contained 10 µM taxol in 1% DMSO.

The flow cell was sealed with VALAP and placed on the TIRF microscope stage. Two-color images were acquired at 2-s intervals and an exposure time of 150 ms. The microscope chamber was heated to ∼35–37°C before image acquisition. Image acquisition was controlled using NIS-Elements AR 4.60.00 (Nikon). Experiments with γ-TuRC^γ-tub-WT^ and γ-TuRC^γ-tubΔGTP^ were performed on the same days using the same reagents.

### Analysis of γ-TuRC capping at stabilized microtubule minus-ends

The number of capped minus-ends was manually counted and then divided by the total number of microtubules within a field of view at ∼3 min after the final reaction mixture was added to the flow cell. The microtubules were observed up to 20 s before and after 3 min to determine the directional movement of the microtubules. Bundled microtubules whose ends could not be determined were excluded from the analysis.

Kymographs were generated using the KymoResliceWide v.0.5 plugin for ImageJ (https://github.com/ekatrukha/KymoResliceWide).

### Purification of recombinant γ-tubulin proteins

pACEBAC1–γ-tubulin–TEV–HIS6 WT or N229A plasmids were transformed into DH10MultiBacTurbo cells, isolated, and transfected into Sf9 cells according per the Bac-to-Bac manual (Invitrogen). Baculoviruses were amplified twice, and fresh P3 virus was used to infect 2 liters of High Five cells (Thermo Fisher Scientific) at a 1:75 dilution and a cell density of 2.5 × 10^6^/ml for 60 h at 27°C. Purification of γ-tubulin was performed as described previously (Rice et al., 2008).

### Quantitative Western blotting

γ-TuRC concentrations were estimated using quantitative Western blotting against purified γ-tubulin. Serial dilutions of γ-tubulin were used to calculate a standard curve, from which the concentration of γ-tubulin in each γ-TuRC was calculated. As each complex contains 14 γ-tubulins, the derived γ-tubulin concentration was then divided by 14 to calculate the final γ-TuRC concentration. Densitometry analysis was performed using the standard features in FIJI.

### Native mass spectrometry (nMS) analysis

The purified protein samples were buffer-exchanged into nMS solution (500 mM ammonium acetate, 0.01% Tween-20) using Zeba desalting microspin columns with a 40 kD molecular weight cut-off (Thermo Scientific). For nucleotide incubation, the buffer-exchanged sample was incubated with fivefold molar excess of Mg-GTP (1 µM protein: 5–10 µM Mg-GTP) on ice for 2 min prior to nMS analysis. Each nMS sample was loaded into a gold-coated quartz capillary tip that was prepared in-house and electrosprayed into an Exactive Plus EMR instrument (Thermo Fisher Scientific) using a modified static nanospray source (Olinares and Chait, 2020). The MS parameters used included: spray voltage, 1.22 kV; capillary temperature, 150°C; S-lens RF level, 200; resolving power, 8,750 at *m/z* of 200; AGC target, 1 × 10^6^; number of microscans, 5; maximum injection time, 200 ms; in-source dissociation (ISD), 10 V; injection flatapole, 8 V; interflatapole, 4 V; bent flatapole, 4 V; high energy collision dissociation (HCD), 125 V; ultrahigh vacuum pressure, 4.5 × 10^−10^ mbar; and total number of scans, 100. Mass calibration in positive EMR mode was performed using cesium iodide. Raw nMS spectra were visualized using Thermo Xcalibur Qual Browser (version 4.2.47). Data processing and spectra deconvolution were performed using UniDec version 4.2.0 (Marty et al., 2015; Reid et al., 2019). The UniDec parameters used were m/z range: 2,000–7,000; mass range: 10,000–200,000 Da; sample mass every 1 Da; smooth charge state distribution, on; peak shape function, Gaussian; and Beta softmax function setting, 20. The expected masses for the wild-type and GTP-deficient mutant (N229A) γ-tubulin with N-terminal methionine removed were 52,713 and 52,670 Da, respectively. The measured masses for these proteins were within ± 2 Da from the expected mass.

### Negative stain EM

Purified γ-TuRC^γ-tubΔGTP^ was applied to glow-discharged carbon-coated copper grids (EMS; CF-400-Cu) and incubated for 1 min per application at RT. The protein solution was removed by manual blotting with Whatman No. 1 filter paper. The application was repeated as necessary to improve particle density depending on the concentration of γ-TuRC. The final application was not blotted off. Freshly filtered 1% uranyl acetate (wt/vol) was then applied to exchange the solution and then incubated on the grid for 1 minute. The grid was blotted to remove the stain. The grid was air-dried for at least 24 h in a sealed container with desiccant before imaging.

Particles on grids were imaged and processed as described previously (Wieczorek et al., 2021). In brief, ∼300,000 autopicked particles were binned by two and subjected to reference-free 2D classification to remove particles likely corresponding to contaminants. Next, a random subset of these particles was used to generate an ab initio model. Then, the ab initio model was used as a reference to perform a 3D auto-refinement step. Re-extracted particles were used to generate four 3D classes. Approximately, 4,300 particles in the 3D class with the highest level of detail were re-extracted from CTF-corrected micrographs at the unbinned pixel size (3.036 Å) and subjected to a final round of 3D autorefinement using one of the 3D classes as a reference model. A composite model of the native human γ-TuRC was then rigid body–fitted into density maps using the “Fit in map” function of Chimera. Protein models were generated using UCSF Chimera (Pettersen et al., 2004) or UCSF ChimeraX (Goddard et al., 2018).

### Liquid chromatography-mass spectrometry

40 µl of γ-TuRC^γ-tubΔGTP^ in 1× SDS sample buffer was loaded on a 10-well, 4–20% Tris-glycine precast gel with “wide wells” (Novex). A current of 150 V was applied until the sample migrated ∼1 cm into the gel. An ∼1 cm × 1 cm gel “plug” was cut, and it was further cut into ∼1-mm cubes. Proteins in the gel were reduced and alkylated and then digested with trypsin (Promega) and lysC (Wako). The generated peptides were analyzed using an Orbitrap Fusion Lumos LC-MS/MS using a 70-min gradient on a pulled-emitter column. The mass spectrometer was operated in high resolution/high mass acquisition mode.

Mass spectrometry data were processed and searched using Proteome Discoverer/Mascot with the Swissprot database.

### Immunofluorescence

Cells were grown on presterilized 12-mm-diameter glass coverslips (Thermo Fisher Scientific). For fixation, the coverslips were transferred into Fixation Buffer (80 mM K-Pipes, pH 6.8, 0.8 mM MgCl_2_, 0.8 mM EDTA, 0.5% Triton X-100, 0.1% glutaraldehyde, and 3.2% paraformaldehyde) prewarmed to 37°C for 20 min. The cells were incubated in Fixation Buffer at 37°C for 10 min and then reduced in 10 mM NaBH_4_ (dissolved in water for) 5 min at RT. After one wash with PBS, the cells were incubated in blocking buffer (1× PBS, 3% BSA, 0.5% Triton X-100, and 0.05% NaN_3_) for 1.5 h at RT. Coverslips were then incubated with primary antibody diluted in Blocking Buffer for 1.5 h at RT. After three washes with 1 × PBS for 5 min at RT, the coverslips were incubated for 1.5 h at RT with secondary antibodies diluted in blocking buffer. After three washes in 1xPBS for 5 min, the coverslips were mounted (20 mM Tris-HCl, pH 8, 0.5% propyl gallate, and 90% glycerol) and sealed with nail polish.

### Cell lysate preparation and Western blotting

Cell lysates were prepared in lysis buffer (25 mM Tris-HCL, pH 7.4, 0.5% IGEPAL-CA-630, 100 mM NaCl, 5 mM MgCl_2_, 20 mM β-glycerophasphate, and 1 mM EDTA) supplemented with 1 mM DTT, 1× cOmplete EDTA-free protease inhibitors (Roche), and 1 mM PMSF. Total lysate concentration was measured by Bradford assay and adjusted with lysis buffer. Lysates were denatured in sample buffer and boiled at 95°C for 10 min. Lysates were run on 4–20% Tris-glycine precast gels at 185V until the sample buffer reached the bottom of the gel. Proteins were then transferred onto Immobilon-P PVDF 0.45 µm membrane (IPVH00010; Merck Millipore) using a wet-tank transfer system (Bio-Rad) in Tris-Glycine transfer buffer (25 mM Tris-HCl, 175 mM Glycine, 20% MeOH). The membrane was blocked in intercept (TBS) blocking buffer (LICOR) for 45 min and incubated in primary antibody diluted in antibody buffer (1xTBS, 5% BSA, 0.02% NaN_3_) overnight at 4°C. Membranes were then washed 3×, 5 min per wash, in 1 × TBS-T. Membranes were then incubated in secondary antibody and diluted in antibody buffer, for 1 h. The membrane was again washed 3 × 5 min per wash in 1 × TBS-T before imaging on the Odyssey imaging system (LICOR). Densitometry was performed using ImageJ. Densitometry was restricted to a comparison of lanes from the same exposure and run on the same gel. The intensity was normalized to loading control.

### Sucrose gradient centrifugation of whole cell lysates

3.5 ml sucrose gradients were prepared as follows: 500 μl of 5, 10.85, 16.7, 22.72, 28.3, 34.15, and 40% sucrose (wt/vol) in gradient buffer (40 mM HEPES, pH 7.5, 150 mM KCl, 1 mM MgCl_2_, 0.01% Tween-20 [vol/vol], 0.1 mM MgGTP, and 1 mM 2-mercaptoethanol) were layered into a 5-ml centrifuge tube using a cut-off P1000 tip. The gradient was allowed to equilibrate overnight at 4°C.

150 μl of cell lysate at a concentration of 2 μg/ml was layered at the top of the gradient. The gradient was centrifuged at 55,000 rpm in a SW-55 rotor at 4°C for 3.5 h with minimum acceleration and no break. 16 × ∼220 µl fractions were manually collected by inserting a needle into the bottom of the gradient and drawing the sample up using a peristaltic pump. Fractions were denatured in 5× sample buffer and boiled at 95°C for 10 min. Samples from each fraction were run on NuPAGE 4–20% Bis-Tris 17-well precast gels at 200 V using NuPAGE MOPS SDS running buffer until the sample buffer reached the bottom of the gel. Proteins were then transferred onto Immobilon-P PVDF 0.45 µm membrane (IPVH00010; Merck Millipore) using a wet-tank transfer system (Bio-Rad) in NuPAGE transfer buffer (Invitrogen). The membrane was processed as described above before imaging on the ChemiDoc Imaging System (Bio-Rad). Densitometry was performed using ImageJ.

For quantitative analysis, the signal intensity of γ-tubulin in each fraction was divided by the sum of the signal intensities of γ-tubulin in all of the fractions.

### Fixed cell microtubule regrowth assay

Cells were grown on presterilized 12-mm-diameter glass coverslips (Thermo Fisher Scientific) in a 10-cm tissue culture dish. Coverslips for individual timepoints were taken from the same culture dish. One coverslip for each experiment was fixed in fixation buffer (described above) prior to nocodazole treatment to confirm the phenotype of the cell sample. To minimize the loss of mitotic cells, coverslips were transferred to new dishes containing the indicated buffers. First, coverslips were incubated in 10 µM nocodazole diluted in DMEM +10% FBS +1 µg/ml Dox for 1 h at 37°C. Coverslips were then transferred to an ice block for an additional 1 h of incubation. Coverslips were then washed 4×, 15 s per wash, in warm (37°C) wash media (DMEM + 10% FBS + 1 µg/ml Dox + 0.1% DMSO), after which the coverslips were incubated at 37°C, followed by fixation at the indicated time points. One coverslip for each experiment was fixed prior to the wash step to confirm complete microtubule depolymerization (indicated as time point 0 in Fig. S2 I). Cells were processed for immunofluorescence as described above.

Western blot analysis of cells processed for microtubule regrowth assays confirmed that nocodazole treatment and ice incubation did not affect the expression patterns of the shRNA targeting endogenous γ-tubulin or of γ-tubulin-GFP (WT and ΔGTP).

### Live cell microtubule regrowth assay

Cells were grown on presterilized 22 × 22-mm square glass coverslips (Thermo Fisher Scientific) in a 10-cm tissue culture dish. To minimize the loss of mitotic cells, coverslips were transferred to new dishes containing the indicated buffers. First, coverslips were incubated in 10 µM nocodazole diluted in DMEM + 10% FBS + 1 µg/ml Dox for 1 h at 37°C. Coverslips were then washed 4×, 15 s per wash, in cold (4°C) wash media (Leibovitz’s L-15 (Gibco) + 10% FBS + 1 µg/ml Dox + 0.1% DMSO). Coverslips were then incubated on an ice block in cold (0°C) Leibovitz’s L-15 media + 1 µg/ml Dox + 100 nM SiR–tubulin (Cytoskeleton) for 1 h. Coverslips were then assembled in a custom Rose chamber in warm (37°C) Leibovitz’s L-15 media + 10% FBS + 1 µg/ml Dox + 100 nM SiR–tubulin. Imaging was initiated within 3 min after the coverslips were removed from ice incubation.

Imaging was performed on a Nikon Eclipse Ti2-E equipped with a Yokogawa W1 confocal scanning unit, a z piezo stage, and a 100× oil objective (Plan Apo, 1.45 NA). The microscope was fitted with an environmental chamber enclosure and heated to 37°C with the AirTherm SMT heating system (WPI). Fluorescence from the GFP and SiR–tubulin channels was excited with a 100 mW 488 nm (Coherent) and 75 mW 640 nm (Coherent) laser, respectively. Both lasers were transmitted to the sample using a custom Yokogawa quad notch filter (405-480-561-640) and fluorescence was filtered using an ET 520/40 m (Chroma Technology) and ET 670/50 m (Chroma Technology) for the GFP and SiR–tubulin channels, respectively. Fluorescence was directed onto a Prime 95B sCMOS camera (Photometrics) and images were recorded using NIS-Elements software (Nikon). The positions of several cells on a coverslip were recorded by imaging with DIC, and these cells were imaged every 5 min. With 0.3-µm spacing between Z-planes, images were taken through 5 µm in the center of the cell, with 50-ms exposure in the GFP channel, and 150-ms exposure in the SiR–tubulin channel. DIC images were used to align cells throughout each time point.

### Counting microtubule foci

The raw images of cells undergoing live microtubule regrowth were compiled as MIPs. To quantify the number of microtubule foci, the following standard FIJI tools were applied. First, a signal intensity threshold of >90% was applied, followed by binarization of the image. The signal was then segmented using the Watershed plugin. A segmented particle greater than 1 µm^2^ was counted as a microtubule foci.

### Statistical analyses

The mean residence times and 95% confidence intervals of the γ-TuRC on dynamic microtubule minus-ends (shown in Fig. 1, J and K; and Fig. 3, E and F) were calculated using MatLab (Chen et al., 2014). All other analyses were performed using the standard features in GraphPad Prism. To calculate the significant difference in the γ-TuRC landing rates on dynamic microtubules (Fig. S1 E), the data were determined to be normal using a D’Agostino and Pearson test, and then a two-sided Student’s *t* test was calculated.

### Online supplemental material

Fig. S1 shows native mass spectrometry analysis of γ-tubulin^N229A^ and further analysis of γ-TuRC^γ-TubΔGTP^. Fig. S2 shows characterization of γ-tubulin cell lines and microtubule regrowth assay in fixed cells. Fig. S3 shows analysis of live cell microtubule regrowth assays.

## Supplementary Material

SourceData F2 is the source file for Fig. 2.

SourceData FS1 is the source file for Fig. S1.

SourceData FS2 is the source file for Fig. S2.

## References

[bib65] Akhmanova, A., and L. Kapitein. 2022. Mechanisms of microtubule organization in differentiated animal cells. Nature Reviews Molecular Cell Biology. 23:541–558. 10.1038/s41580-022-00473-y 35383336

[bib1] Aldaz, H., L.M. Rice, T. Stearns, and D.A. Agard. 2005. Insights into microtubule nucleation from the crystal structure of human γ-tubulin. Nature. 435:523–527. 10.1038/nature03586 15917813

[bib2] Baumgart, J., M. Kraus, M. Ziehm, M. Kirchner, M. Schafstedde, M. Kelm, S. Niquet, N.M. Stephen, I. Baczko, C. Knosalla, . 2019. Soluble tubulin is significantly enriched at mitotic centrosomes. J Cell Biol. 218:3977–3985. 10.1083/jcb.201902069 31636117 PMC6891098

[bib3] Brilot, A.F., A.S. Lyon, A. Zelter, S. Viswanath, A. Maxwell, M.J. MacCoss, E.G. Muller, A. Sali, T.N. Davis, and D.A. Agard. 2021. CM1-driven assembly and activation of yeast γ-tubulin small complex underlies microtubule nucleation. Elife. 10:e65168. 10.7554/eLife.65168 33949948 PMC8099430

[bib4] Chen, Y., N.C. Deffenbaugh, C.T. Anderson, and W.O. Hancock. 2014. Molecular counting by photobleaching in protein complexes with many subunits: Best practices and application to the cellulose synthesis complex. Mol. Biol. Cell. 25:3630–3642. 10.1091/mbc.e14-06-1146 25232006 PMC4230622

[bib5] Choi, Y.-K., P. Liu, S.K. Sze, C. Dai, and R.Z. Qi. 2010. CDK5RAP2 stimulates microtubule nucleation by the γ-tubulin ring complex. J. Cell Biol. 191:1089–1095. 10.1083/jcb.201007030 21135143 PMC3002024

[bib6] Consolati, T., J. Locke, J. Roostalu, Z.A. Chen, J. Gannon, J. Asthana, W.M. Lim, F. Martino, M.A. Cvetkovic, J. Rappsilber, . 2020. Microtubule nucleation properties of single human γTuRCs explained by their cryo-EM structure. Dev. Cell. 53:603–617.e8. 10.1016/j.devcel.2020.04.019 32433913 PMC7280788

[bib7] Cota, R.R., N. Teixidó-Travesa, A. Ezquerra, S. Eibes, C. Lacasa, J. Roig, and J. Lüders. 2017. MZT1 regulates microtubule nucleation by linking γTuRC assembly to adapter-mediated targeting and activation. J. Cell Sci. 130:406–419 27852835 10.1242/jcs.195321

[bib8] David, A.F., P. Roudot, W.R. Legant, E. Betzig, G. Danuser, and D.W. Gerlich. 2019. Augmin accumulation on long-lived microtubules drives amplification and kinetochore-directed growth. J. Cell Biol. 218:2150–2168. 10.1083/jcb.201805044 31113824 PMC6605806

[bib9] Gaglio, T., A. Saredi, and D.A. Compton. 1995. NuMA is required for the organization of microtubules into aster-like mitotic arrays. J. Cell Biol. 131:693–708. 10.1083/jcb.131.3.693 7593190 PMC2120610

[bib10] Ginsburg, A., A. Shemesh, A. Millgram, R. Dharan, Y. Levi-Kalisman, I. Ringel, and U. Raviv. 2017. Structure of dynamic, taxol-stabilized, and GMPPCP-stabilized microtubule. J. Phys. Chem. B. 121:8427–8436. 10.1021/acs.jpcb.7b01057 28820593

[bib11] Goddard, T.D., C.C. Huang, E.C. Meng, E.F. Pettersen, G.S. Couch, J.H. Morris, and T.E. Ferrin. 2018. UCSF ChimeraX: Meeting modern challenges in visualization and analysis. Protein Sci. 27:14–25. 10.1002/pro.3235 28710774 PMC5734306

[bib12] Gombos, L., A. Neuner, M. Berynskyy, L.L. Fava, R.C. Wade, C. Sachse, and E. Schiebel. 2013. GTP regulates the microtubule nucleation activity of γ-tubulin. Nat. Cell Biol. 15:1317–1327. 10.1038/ncb2863 24161932

[bib13] Gruss, O.J., M. Wittmann, H. Yokoyama, R. Pepperkok, T. Kufer, H. Silljé, E. Karsenti, I.W. Mattaj, and I. Vernos. 2002. Chromosome-induced microtubule assembly mediated by TPX2 is required for spindle formation in HeLa cells. Nat. Cell Biol. 4:871–879. 10.1038/ncb870 12389033

[bib14] Hallen, M.A., J. Ho, C.D. Yankel, and S.A. Endow. 2008. Fluorescence recovery kinetic analysis of gamma-tubulin binding to the mitotic spindle. Biophys. J. 95:3048–3058. 10.1529/biophysj.108.134593 18567627 PMC2527240

[bib15] Hannak, E., K. Oegema, M. Kirkham, P. Gönczy, B. Habermann, and A.A. Hyman. 2002. The kinetically dominant assembly pathway for centrosomal asters in Caenorhabditis elegans is γ-tubulin dependent. J. Cell Biol. 157:591–602. 10.1083/jcb.200202047 12011109 PMC2173857

[bib16] Haren, L., M.H. Remy, I. Bazin, I. Callebaut, M. Wright, and A. Merdes. 2006. NEDD1-dependent recruitment of the gamma-tubulin ring complex to the centrosome is necessary for centriole duplication and spindle assembly. J. Cell Biol. 172:505–515. 10.1083/jcb.200510028 16461362 PMC2063671

[bib17] Haren, L., D. Farache, L. Emorine, and A. Merdes. 2020. A stable sub-complex between GCP4, GCP5 and GCP6 promotes the assembly of γ-tubulin ring complexes. J. Cell Sci. 133. 10.1242/jcs.244368 32317396

[bib18] Heald, R., and A. Khodjakov. 2015. Thirty years of search and capture: The complex simplicity of mitotic spindle assembly. J. Cell Biol. 211:1103–1111. 10.1083/jcb.201510015 26668328 PMC4687881

[bib19] Heald, R., R. Tournebize, T. Blank, R. Sandaltzopoulos, P. Becker, A. Hyman, and E. Karsenti. 1996. Self-organization of microtubules into bipolar spindles around artificial chromosomes in Xenopus egg extracts. Nature. 382:420–425. 10.1038/382420a0 8684481

[bib20] Hutchins, J.R.A., Y. Toyoda, B. Hegemann, I. Poser, J.K. Hériché, M.M. Sykora, M. Augsburg, O. Hudecz, B.A. Buschhorn, J. Bulkescher, . 2010. Systematic analysis of human protein complexes identifies chromosome segregation proteins. Science. 328:593–599. 10.1126/science.1181348 20360068 PMC2989461

[bib21] Jiang, K., L. Rezabkova, S. Hua, Q. Liu, G. Capitani, A.F.M. Altelaar, A.J.R. Heck, R.A. Kammerer, M.O. Steinmetz, and A. Akhmanova. 2017. Microtubule minus-end regulation at spindle poles by an ASPM-katanin complex. Nat. Cell Biol. 19:480–492. 10.1038/ncb3511 28436967 PMC5458804

[bib22] Kapoor, T.M. 2017. Metaphase spindle assembly. Biology. 6:8. 10.3390/biology6010008 28165376 PMC5372001

[bib23] Kapust, R.B., J. Tözsér, J.D. Fox, D.E. Anderson, S. Cherry, T.D. Copeland, and D.S. Waugh. 2001. Tobacco etch virus protease: Mechanism of autolysis and rational design of stable mutants with wild-type catalytic proficiency. Protein Eng. 14:993–1000. 10.1093/protein/14.12.993 11809930

[bib24] Kollman, J.M., J.K. Polka, A. Zelter, T.N. Davis, and D.A. Agard. 2010. Microtubule nucleating γ-TuSC assembles structures with 13-fold microtubule-like symmetry. Nature. 466:879–882. 10.1038/nature09207 20631709 PMC2921000

[bib25] Lecland, N., and J. Lüders. 2014. The dynamics of microtubule minus ends in the human mitotic spindle. Nat. Cell Biol. 16:770–778. 10.1038/ncb2996 24976384

[bib26] Liu, P., E. Zupa, A. Neuner, A. Böhler, J. Loerke, D. Flemming, T. Ruppert, T. Rudack, C. Peter, C. Spahn, . 2020. Insights into the assembly and activation of the microtubule nucleator γ-TuRC. Nature. 578:467–471. 10.1038/s41586-019-1896-6 31856152

[bib27] Lüders, J., U.K. Patel, and T. Stearns. 2006. GCP-WD is a γ-tubulin targeting factor required for centrosomal and chromatin-mediated microtubule nucleation. Nat. Cell Biol. 8:137–147. 10.1038/ncb1349 16378099

[bib29] Lukinavičius, G., L. Reymond, E. D’Este, A. Masharina, F. Göttfert, H. Ta, A. Güther, M. Fournier, S. Rizzo, H. Waldmann, . 2014. Fluorogenic probes for live-cell imaging of the cytoskeleton. Nat. Methods. 11:731–733. 10.1038/nmeth.2972 24859753

[bib30] Mahoney, N.M., G. Goshima, A.D. Douglass, and R.D. Vale. 2006. Making microtubules and mitotic spindles in cells without functional centrosomes. Curr. Biol. 16:564–569. 10.1016/j.cub.2006.01.053 16546079

[bib31] Martin, M., and A. Akhmanova. 2018. Coming into focus: Mechanisms of microtubule minus-end organization. Trends Cell Biol. 28:574–588. 10.1016/j.tcb.2018.02.011 29571882

[bib32] Marty, M.T., A.J. Baldwin, E.G. Marklund, G.K. Hochberg, J.L. Benesch, and C.V. Robinson. 2015. Bayesian deconvolution of mass and ion mobility spectra: From binary interactions to polydisperse ensembles. Anal. Chem. 87:4370–4376. 10.1021/acs.analchem.5b00140 25799115 PMC4594776

[bib33] McKinley, K.L., and I.M. Cheeseman. 2017. Large-scale analysis of CRISPR/Cas9 cell-cycle knockouts reveals the diversity of p53-dependent responses to cell-cycle defects. Dev. Cell. 40:405–420.e2. 10.1016/j.devcel.2017.01.012 28216383 PMC5345124

[bib34] Merdes, A., K. Ramyar, J.D. Vechio, and D.W. Cleveland. 1996. A complex of NuMA and cytoplasmic dynein is essential for mitotic spindle assembly. Cell. 87:447–458. 10.1016/S0092-8674(00)81365-3 8898198

[bib35] Meunier, S., and I. Vernos. 2016. Acentrosomal microtubule assembly in mitosis: The where, when, and how. Trends Cell Biol. 26:80–87. 10.1016/j.tcb.2015.09.001 26475655

[bib36] Murphy, S.M., A.M. Preble, U.K. Patel, K.L. O’Connell, D.P. Dias, M. Moritz, D. Agard, J.T. Stults, and T. Stearns. 2001. GCP5 and GCP6: Two new members of the human gamma-tubulin complex. Mol. Biol. Cell. 12:3340–3352. 10.1091/mbc.12.11.3340 11694571 PMC60259

[bib37] Needleman, D.J., A. Groen, R. Ohi, T. Maresca, L. Mirny, and T. Mitchison. 2010. Fast microtubule dynamics in meiotic spindles measured by single molecule imaging: Evidence that the spindle environment does not stabilize microtubules. Mol. Biol. Cell. 21:323–333. 10.1091/mbc.e09-09-0816 19940016 PMC2808228

[bib38] Oegema, K., C. Wiese, O.C. Martin, R.A. Milligan, A. Iwamatsu, T.J. Mitchison, and Y. Zheng. 1999. Characterization of two related Drosophila γ-tubulin complexes that differ in their ability to nucleate microtubules. J. Cell Biol. 144:721–733. 10.1083/jcb.144.4.721 10037793 PMC2132928

[bib39] Olinares, P.D.B., and B.T. Chait. 2020. Native mass spectrometry analysis of affinity-captured endogenous yeast RNA exosome complexes. Methods Mol. Biol. 2062:357–382. 10.1007/978-1-4939-9822-7_17 31768985

[bib40] Pettersen, E.F., T.D. Goddard, C.C. Huang, G.S. Couch, D.M. Greenblatt, E.C. Meng, and T.E. Ferrin. 2004. UCSF Chimera--a visualization system for exploratory research and analysis. J. Comput. Chem. 25:1605–1612. 10.1002/jcc.20084 15264254

[bib41] Rai, A., T. Liu, E.A. Katrukha, J. Estévez-Gallego, S.W. Manka, I. Paterson, J.F. Díaz, L.C. Kapitein, C.A. Moores, and A. Akhmanova. 2021. Lattice defects induced by microtubule-stabilizing agents exert a long-range effect on microtubule growth by promoting catastrophes. Proc. Natl. Acad. Sci. USA. 118:e2112261118. 10.1073/pnas.2112261118 34916292 PMC8713758

[bib42] Reid, D.J., J.M. Diesing, M.A. Miller, S.M. Perry, J.A. Wales, W.R. Montfort, and M.T. Marty. 2019. MetaUniDec: High-Throughput deconvolution of native mass spectra. J. Am. Soc. Mass Spectrom. 30:118–127. 10.1007/s13361-018-1951-9 29667162 PMC6192864

[bib43] Renda, F., C. Miles, I. Tikhonenko, R. Fisher, L. Carlini, T.M. Kapoor, A. Mogilner, and A. Khodjakov. 2022. Non-centrosomal microtubules at kinetochores promote rapid chromosome biorientation during mitosis in human cells. Curr. Biol. 32:1049–1063.e4. 10.1016/j.cub.2022.01.013 35108523 PMC8930511

[bib44] Rice, L.M., E.A. Montabana, and D.A. Agard. 2008. The lattice as allosteric effector: Structural studies of alphabeta- and γ-tubulin clarify the role of GTP in microtubule assembly. Proc. Natl. Acad. Sci. USA. 105:5378–5383. 10.1073/pnas.0801155105 18388201 PMC2291134

[bib45] Romberg, L., D.W. Pierce, and R.D. Vale. 1998. Role of the kinesin neck region in processive microtubule-based motility. J. Cell Biol. 140:1407–1416. 10.1083/jcb.140.6.1407 9508773 PMC2132664

[bib46] Sanchez, A.D., and J.L. Feldman. 2017. Microtubule-organizing centers: From the centrosome to non-centrosomal sites. Curr. Opin. Cell Biol. 44:93–101. 10.1016/j.ceb.2016.09.003 27666167 PMC5362366

[bib47] Strome, S., J. Powers, M. Dunn, K. Reese, C.J. Malone, J. White, G. Seydoux, and W. Saxton. 2001. Spindle dynamics and the role of γ-tubulin in early Caenorhabditis elegans embryos. Mol. Biol. Cell. 12:1751–1764. 10.1091/mbc.12.6.1751 11408582 PMC37338

[bib48] Thevenaz, P., U.E. Ruttimann and M. Unser. 1998. A pyramid approach to subpixel registration based on intensity. IEEE Trans Image Process. 7:27–41. 10.1109/83.650848 18267377

[bib49] Ti, S.C., M.C. Pamula, S.C. Howes, C. Duellberg, N.I. Cade, R.E. Kleiner, S. Forth, T. Surrey, E. Nogales, and T.M. Kapoor. 2016. Mutations in human tubulin proximal to the kinesin-binding site alter dynamic instability at microtubule plus- and minus-ends. Dev. Cell. 37:72–84. 10.1016/j.devcel.2016.03.003 27046833 PMC4832424

[bib50] Tsuchiya, K., and G. Goshima. 2021. Microtubule-associated proteins promote microtubule generation in the absence of γ-tubulin in human colon cancer cells. J. Cell Biol. 220:e202104114. 10.1083/jcb.202104114 34779859 PMC8598081

[bib51] Tulu, U.S., C. Fagerstrom, N.P. Ferenz, and P. Wadsworth. 2006. Molecular requirements for kinetochore-associated microtubule formation in mammalian cells. Curr. Biol. 16:536–541. 10.1016/j.cub.2006.01.060 16527751 PMC1500889

[bib52] Uphoff, C.C. and H.G. Drexler. 2014. Detection of mycoplasma contamination in cell cultures. Curr. Protoc. Mol. Biol. 106:28.4.1–14. 10.1002/0471142727.mb2804s106 24733240

[bib53] Walker, R.A., E.T. O’Brien, N.K. Pryer, M.F. Soboeiro, W.A. Voter, H.P. Erickson, and E.D. Salmon. 1988. Dynamic instability of individual microtubules analyzed by video light microscopy: Rate constants and transition frequencies. J. Cell Biol. 107:1437–1448. 10.1083/jcb.107.4.1437 3170635 PMC2115242

[bib54] Watanabe, S., F. Meitinger, A.K. Shiau, K. Oegema, and A. Desai. 2020. Centriole-independent mitotic spindle assembly relies on the PCNT-CDK5RAP2 pericentriolar matrix. J. Cell Biol. 219:e202006010. 10.1083/jcb.202006010 33170211 PMC7658699

[bib55] Wieczorek, M., L. Urnavicius, S.C. Ti, K.R. Molloy, B.T. Chait, and T.M. Kapoor. 2020a. Asymmetric molecular architecture of the human γ-tubulin ring complex. Cell. 180:165–175.e16. 10.1016/j.cell.2019.12.007 31862189 PMC7027161

[bib56] Wieczorek, M., T.-L. Huang, L. Urnavicius, K.C. Hsia, and T.M. Kapoor. 2020b. MZT proteins form multi-faceted structural modules in the γ-tubulin ring complex. Cell Rep. 31:107791. 10.1016/j.celrep.2020.107791 32610146 PMC7416306

[bib57] Wieczorek, M. S.C. Ti, L. Urnavicius, K.R. Molloy, A. Aher, B.T. Chait, and T.M. apoor. 2021. Biochemical reconstitutions reveal principles of human γ-TuRC assembly and function. J. Cell Biol. 220:e202009146. 10.1083/jcb.202009146 33496729 PMC7844428

[bib58] Wiese, C., and Y. Zheng. 2000. A new function for the γ-tubulin ring complex as a microtubule minus-end cap. Nat. Cell Biol. 2:358–364. 10.1038/35014051 10854327

[bib59] Woodruff, J.B., B. Ferreira Gomes, P.O. Widlund, J. Mahamid, A. Honigmann, and A.A. Hyman. 2017. The centrosome is a selective condensate that nucleates microtubules by concentrating tubulin. Cell. 169:1066–1077.e10. 10.1016/j.cell.2017.05.028 28575670

[bib60] Würtz, M., A. Böhler, A. Neuner, E. Zupa, L. Rohland, P. Liu, B.J.A. Vermeulen, S. Pfeffer, S. Eustermann, and E. Schiebel. 2021. Reconstitution of the recombinant human γ-tubulin ring complex. Open Biol. 11:200325. 10.1098/rsob.200325 33529551 PMC8061689

[bib61] Würtz, M., E. Zupa, E.S. Atorino, A. Neuner, A. Böhler, A.S. Rahadian, B.J.A. Vermeulen, G. Tonon, S. Eustermann, E. Schiebel, and S. Pfeffer. 2022. Modular assembly of the principal microtubule nucleator γ-TuRC. Nat. Commun. 13:473. 10.1038/s41467-022-28079-0 35078983 PMC8789826

[bib62] Young, A., J.B. Dictenberg, A. Purohit, R. Tuft, and S.J. Doxsey. 2000. Cytoplasmic dynein-mediated assembly of pericentrin and gamma tubulin onto centrosomes. Mol. Biol. Cell. 11:2047–2056. 10.1091/mbc.11.6.2047 10848628 PMC14902

[bib63] Zheng, Y., M.L. Wong, B. Alberts, and T. Mitchison. 1995. Nucleation of microtubule assembly by a γ-tubulin-containing ring complex. Nature. 378:578–583. 10.1038/378578a0 8524390

[bib64] Zimmermann, F., M. Serna, A. Ezquerra, R. Fernandez-Leiro, O. Llorca, and J. Luders. 2020. Assembly of the asymmetric human γ-tubulin ring complex by RUVBL1-RUVBL2 AAA ATPase. Sci. Adv. 6:1–20. 10.1126/sciadv.abe0894 PMC11206223 33355144

