# Supplementary figures and images for: A nucleotide binding–independent role for γ-tubulin in microtubule capping and cell division
Source: J Cell Biol. 2023 Jan 25;222(3):e202204102. doi: 10.1083/jcb.202204102 (PMC9930161; doi:10.1083/jcb.202204102)

Figure 2A

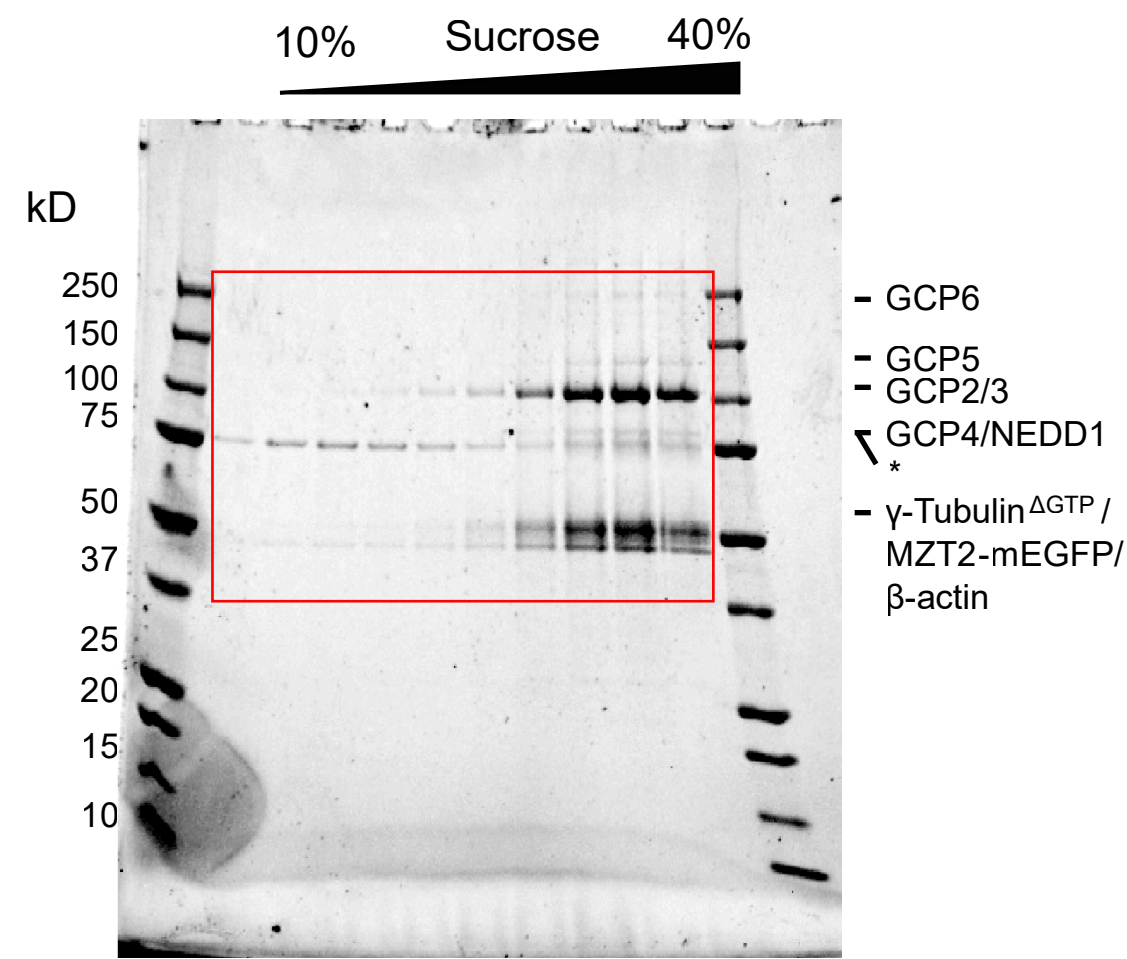

Supplement: SourceData F2 — is the source file for Fig. 2. [file JCB_202204102_SourceDataF2.pdf]

# Source Data Supplementary Figure 1

## Supplementary Figure 1A

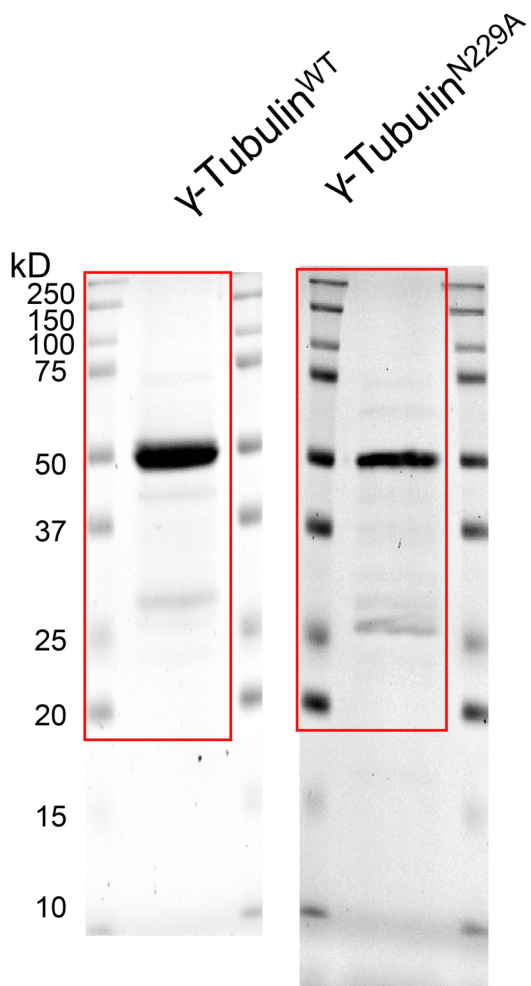

Supplement: SourceData FS1 — is the source file for Fig. S1. [file JCB_202204102_SourceDataFS1.pdf]

## Supplementary Figure 2A

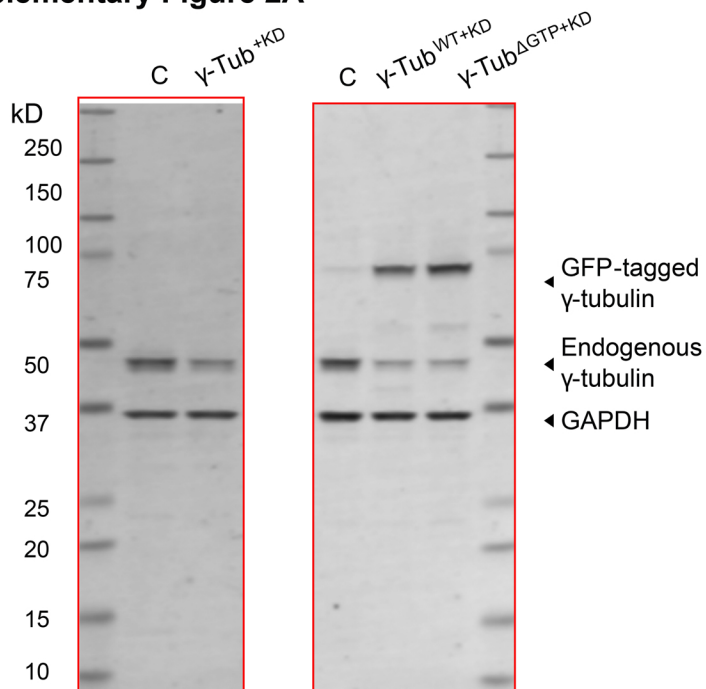

## Supplementary Figure 2G

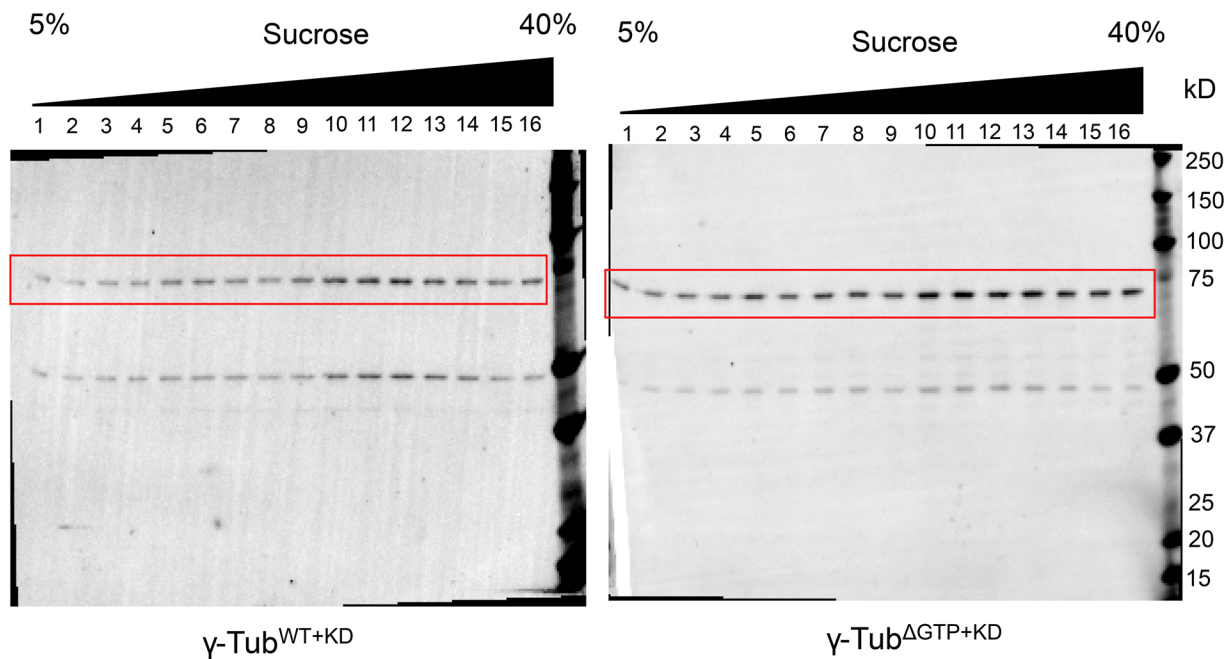

Supplement: SourceData FS2 — is the source file for Fig. S2. [file JCB_202204102_SourceDataFS2.pdf]
